# Does Body Shape in *Fundulus* Adapt to Variation in Habitat Salinity?

**DOI:** 10.3389/fphys.2019.01400

**Published:** 2019-11-15

**Authors:** Joseph M. Styga, Jason Pienaar, Peter A. Scott, Ryan L. Earley

**Affiliations:** ^1^Department of Biological Sciences, The University of Alabama, Tuscaloosa, AL, United States; ^2^Biology Program, Centre College, Danville, KY, United States; ^3^Department of Ecology and Evolutionary Biology, University of California, Los Angeles, Los Angeles, CA, United States

**Keywords:** osmoregulation, geometric morphometrics, body shape, development, *Fundulus*

## Abstract

Understanding the ecological pressures that generate variation in body shape is important because body shape profoundly affects physiology and overall fitness. Using *Fundulus*, a genus of fish that exhibits considerable morphological and physiological variation with evidence of repeated transitions between freshwater and saltwater habitats, we tested whether habitat salinity has influenced the macroevolution of body shape at different stages in development. After accounting for phylogenetic inertia, we find that body shape deviates from the optimal streamlined shape in a manner consistent with different osmoregulatory pressures exerted by different salinity niches at every stage of ontogeny that we examined. We attribute variation in body shape to differential selection for osmoregulatory efficiency because: (1) saline intolerant species developed body shapes with relatively low surface areas more conducive to managing osmoregulatory demands and (2) inland species that exhibit high salinity tolerances have body shapes similar to saline tolerant species in marine environments.

## Introduction

Natural selection is a major driver of the evolution of morphological diversity among species (Arnold, 1983; Schluter and Smith, 1986; Berner et al., 2008). However, other factors, such as genetic drift (Ackermann and Cheverud, 2004) and evolutionary constraints, which prevent traits from reaching their optima (e.g., phylogenetic inertia; Hansen and Orzack, 2005; Pienaar et al., 2013), also have the potential to influence the direction and magnitude of morphological evolution among species. As a result, the relative contribution of these factors needs to be considered when examining the evolution of morphological variation among species (Hansen and Orzack, 2005).

Advances in phylogenetic comparative methods (Hansen, 1997; Butler and King, 2004; Hansen et al., 2008; Bartoszek et al., 2012) have enhanced our ability to identify ecological pressures that underlie morphological variation among taxa, while also accounting for stochastic influences and phylogenetic inertia on current trait values. Numerous studies have identified putative selection pressures associated with morphological evolution in every major vertebrate group, including climate variation and color polymorphism in amphibians (Fisher-Reid and Weins, 2015), foraging behavior and caudal skeleton variation in birds (Felice and O’Connor, 2014), dietary niche and jaw morphology in perciform fishes (Grubich and Grubich, 2003), macrohabitat use and various morphological characters in anoles lizards (Glor et al., 2003), and dietary niche and skull shape in bats (Nogueira et al., 2009). Few studies, however, have examined ecological pressures that might have driven selection for morphological variation at several stages in development and in a phylogenetic context (Harvey and Strand, 2002). Natural selection can act differently throughout ontogeny (Gignac and Santana, 2016). Thus, selection pressures that might seem to be important in influencing variation at one stage of development may not be important at other stages in development. Therefore, to obtain a clearer understanding of how selection might drive morphological variation over macroevolutionary time, it is important to take an ontogenetic perspective.

Furthermore, few studies have investigated the ecological pressures that might have driven selection for ontogenetic differences in complex, high dimensional traits such as body shape (Adams and Nistri, 2010). Understanding the ecological pressures that generate variation in body shape is important because body shape can often have profound effects on all aspects of physiology and fitness. For instance, body shape influences fast-start (predator avoidance) performance in *Gambusia*, which in turn, appears to be driven by selection imposed by predation. Specifically, compared to individuals from low-predation sites, *Gambusia* from high-predation sites possess more robust heads, narrower bodies, and wider caudal regions, all of which are more conducive to better fast-starts (Langerhans et al., 2004). In addition, selection imposed by high temperatures and toxins (e.g., hydrogen sulfide) appears to favor larger head sizes in poeciliid fishes (e.g., *Limia perugiae*, *Poecilia sulphuraria*), which might support greater gill surface area and thereby allow the animals to survive in hypoxic environments (Tobler et al., 2011; Weaver et al., 2016). However, the relationship between body shape and putative ecological pressures typically is only compared among populations or species at the adult stage (Price et al., 2015). As a result, we may not be able to assess the full scope of body shape variation among populations or species that has arisen through natural selection. Thus, our conclusions about the relative importance of an ecological variable in contributing to morphological variation among populations or species may be age-dependent.

One genus of fishes, *Fundulus* (Teleostei: Cyprinoformes: Fundulidae), provides an ideal model in which to conduct such analyses because a single selective pressure, salinity niche, has been hypothesized as a major contributor to the evolutionary diversification of this group (Whitehead, 2010). Most of the 39–44 currently recognized *Fundulus* species have highly variable salinity tolerances (Rodgers et al., 2018). Salinity niche, which we describe as all aspects of a species’ environment that change with salinity, also appears to have driven morphological variation in fish, especially as it relates to osmoregulation, and the gills and kidneys. For instance, selection associated with salinity may have resulted in variation in chloride cell densities between different populations of *Fundulus heteroclitus* (Scott et al., 2004). In addition, selection imposed by different salinity regimes may have resulted in locally adapted (and plastic) kidney morphologies in sticklebacks (Hasan et al., 2017).

Although the influence of salinity niche on body shape is less clear, there are a number of reasons why variation in body shape may also be related to salinity niche among *Fundulus* species. First, salinity niche may influence the type (e.g., gape-limited, ambush) and density of predators encountered by populations of a certain species, which in turn may lead to the evolution of a selectively advantageous body shape within that species. For instance, it is well known that a wide mid-body depth may be selectively advantageous for prey fish because it may prevent mortality imposed by gape-limited predators (Walker, 1997; Walker and Bell, 2000). However, freshwater and brackish *Fundulus* species exhibit similar morphologies in response to predators (Langerhans et al., 2004; Langerhans and Makowicz, 2009), thus we discount this hypothesis as an explanation for variation in *Fundulus* body shape across salinity niches.

Second, because teleosts lose water and ions passively through their skin (Perry, 1997; Karnaky, 1998), natural selection driven by osmoregulatory efficiency may contribute to body shape variation between high and low salinity environments. Freshwater fish are hyperosmotic to their surrounding environment, and thus, must combat the constant influx of water. Although they have many physiological adaptations to counteract this process, freshwater fish can still spend upward of 20–50% of their energy budget on osmoregulation (Boeuf and Payan, 2001). To help minimize solutes or water being passively lost to or gained from the external environment, respectively, selection imposed by osmoregulatory efficiency may have resulted in body shapes with lower surface areas (i.e., more cylindrical, fusiform body shapes) in freshwater *Fundulus*. This type of evolutionary response to freshwater habitats follows from Fick’s law, which states that the rate of diffusion across a surface is directly proportional to the product of its area, permeability, and the concentration gradient. In fishes, the skin is permeable to water (Talbot et al., 1982). If we assume that skin permeability is a constant, body surface area, which is tightly linked with body shape, emerges as a potentially key feature associated with adaptation to various salinity niches. As such, lower surface areas should be favored in habitats, like freshwater, where solute conservation and minimization of water uptake are essential for fitness.

Third, body shape variation in *Fundulus* may be due to an evolutionary constraint associated with gill surface area. Certain body shapes may be more accommodating to gills with smaller or larger surface areas, which either reduce or promote ion exchange, respectively (Jensen et al., 1998). Wider or deeper heads may provide the space necessary for larger gills (also more lamellae and larger densities of chloride cells) in saltwater fish, which must actively and passively transport excess ions across the gills to maintain homeostasis, and in fish that occupy hypoxic environments, where respiratory surface area must be maximized (Jensen et al., 1998; see also Tobler et al., 2011; Weaver et al., 2016). However, bigger heads may also require concomitant changes in the shape of the trunk and tail to sustain effective swimming and feeding.

Finally, flow rate, a major driver of body shape in fish (Meyers and Belk, 2014), may also vary predictably with salinity (i.e., freshwater streams and rivers have higher flow rates than estuaries and salt marshes), and thus, may cause a close association between salinity tolerance and body shape. Despite this, few studies have examined the effect of salinity niche on body shape among species. In fishes, fineness ratio (FR; ratio of body length to body width) is often used to study their hydrodynamics, where a value of 4.5 has been experimentally determined to be the ratio at which drag is minimized and body volume is maximized in a fluid medium (Walker et al., 2013). If drag were the only selection pressure acting on morphology over the evolutionary divergence of *Fundulus*, we would expect the optimum FR for all species to be close to 4.5. However, if other environmental variables are associated with morphological evolution in *Fundulus*, then we would expect FR to deviate from 4.5 in a manner that is predictable given the particular environment that the species inhabits.

Here, we determine if salinity niche (i.e., freshwater/brackish or saltwater) or salinity scope (i.e., “narrow” or “wide” range of salinity tolerance) is associated with morphological variation at three different age groups among *Fundulus* species. We focus on two morphological traits in particular: body shape and FR, which we extract from a geometric morphometric characterization of body shapes. We use various phylogenetic comparative methods to test hypotheses for macroevolution of these traits in relation to salinity. The first set of methods, based on a multivariate Brownian motion (BM) model of evolution can determine if two traits have been correlated over macroevolutionary time scales whilst controlling for statistical phylogenetic effects (Felsenstein, 1985). The second set of methods, based on an Ornstein-Uhlenbeck (OU) process, models adaptation of trait values toward hypothesized niche optima (Hansen, 1997; Butler and King, 2004; Hansen et al., 2008; Bartoszek et al., 2012), and allows us to test for adaptation to the environment. Considering that multiple shifts in salinity niche (i.e., shifts from freshwater to saltwater habitats) have occurred during the evolutionary history of *Fundulus* (Whitehead, 2010), we hypothesized that salinity niche and salinity scope have exerted strong selection on FR within each age group among *Fundulus* species. We demonstrate that FR evolved in response to salinity niche (saltwater vs. brackish/freshwater). We conducted identical analyses using gill arch length (mm) and opercular epithelium surface area (mm^2^) as response variables and found that these were not significantly predicted by salinity niche.

## Materials and Methods

### Sample Specimens

A total of 2,267 specimens from all size classes belonging to 19 *Fundulus* species were obtained from The University of Alabama’s Ichthyological Collection (Supplementary Table S1). Because our research was conducted on previously euthanized, museum specimens (i.e., we did not handle or euthanize any live organisms during the course of our study), this research was exempt from ethics approval. For each species, we assigned each specimen to one of three size classes: “young” (lowest 25% of specimens), “intermediate” (middle 25% of specimens), and “old” (highest 25% of specimens) based on their standard length (SL) (Supplementary Figures S1A–E). Although a number of environmental variables can cause the relationship between size and age to evolve (Angilletta et al., 2004), by comparing morphological variation across such divergent size classes among species, variation in size can likely be attributed to variation in age. For museum specimens, different methods of fixation and preservation can also influence shape variation among species (Martinez et al., 2012). As a result, we focused on incorporating specimens that were fixed and stored using the same procedure (fixed in formalin and stored in 70% ethanol).

Species were classified into two salinity niches (F = freshwater/brackish, or S = saltwater), according to Ghedotti and Davis (2013; see Supplementary Table S1). Species that had a maximum salinity tolerance greater than 25 ppt were classified as saltwater fish while species that had a maximum salinity tolerance less than 25 ppt were classified as freshwater/brackish fish. In addition to a salinity classification, we also included salinity scope, or the difference between a species’ maximum and minimum salinity tolerance, as a dependent variable in our macroevolutionary analyses. We included this variable in our analyses as a way to determine if selection might have favored phenotypic plasticity in our morphological traits. If phenotypic plasticity in response to salinity conditions contributed to morphological evolution, then variation should be concentrated within species and be more related to salinity scope (i.e., within-species variation in salinity tolerance) than salinity niche. We classified species into two salinity scope categories (N = “narrow” or W = “wide”). Species were classified as having narrow scopes if the difference between maximum and minimum salinity tolerances was less than one part per thousand (ppt). If scope were greater than 1 ppt, species were classified as having a wide scope. For some species, population-level differences in salinity tolerance may exist. For instance, *Fundulus chrysotus* from Biloxi, Mississippi are reported to have a salinity tolerance of 60 ppt (Rakocinski et al., 1997), while individuals from the Florida panhandle are reported to have a salinity tolerance of 20.5 ppt (Griffith, 1974). For these species, we ascribed salinity tolerance in accordance with the collection location of our specimens (i.e., we used 20.5 ppt for *F. chrysotus* because our specimens were collected in Florida).

### Geometric Morphometrics

**FIGURE 1:**
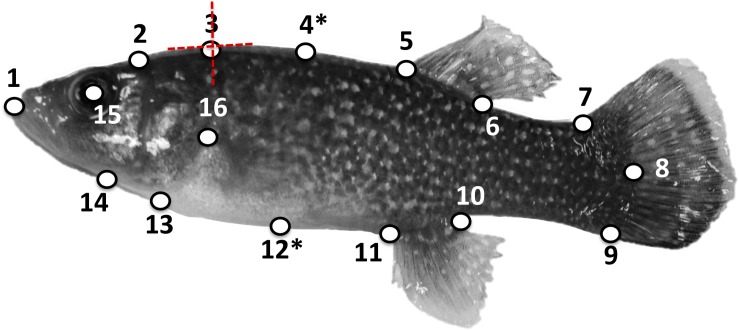
Locations of 16 type II landmarks on a mature *Fundulus grandis* specimen as in Schaefer and Duvernell (2011), except for the removal of the second LM and shift of LM5 to the center of the eye. See text and Supplementary Material for details on the landmarks. Asterisks (^∗^) denote sliding LMs. Each LM was permitted to shift in the adaptive OU models in two dimensions as depicted for LM3.

Each specimen was photographed using a Nikon^®^ D1x camera with a 40 mm microlens in a lateral view for geometric morphometric analysis conducted in the R package geomorph (Adams and Otárola-Castillo, 2013). A total of 16 homologous landmarks (LM) were digitized on each specimen in TpsDig2 (Rohlf, 2001) following a LM configuration slightly modified from Schaefer and Duvernell (2011) (Figure 1 and Supplementary Description A1), and specimens were also scaled to the nearest mm during the digitization process. Prior to further landmark transformations, we calculated FR from our geometric morphometric data by dividing the length (mm) of the specimen by its width (mm). We calculated the length of each specimen (SL) as the distance between LM1 and LM8 using the following formula:

$$\surd \left({\left(X\,1-X\,8\right)}^{2}+{\left(Y\,1-Y\,8\right)}^{2}\right)$$

where *X*1 is the *x*-coordinate of LM1, *X*8 is the *x*-coordinate of LM8, *Y*1 is the *y*-coordinate of LM1, and *Y*8 is the *y*-coordinate of LM8. Using the same equation, we calculated the width of each specimen at its widest point, which corresponded to the distance between LM4 and LM12. We then divided length by width to obtain FR. For FR, we obtained an average for each species. Gill arch length (mm) and opercular epithelium surface area (mm^2^) were also measured. Details of these measurements can be found in Supplementary Description A2.

**FIGURE 2:**
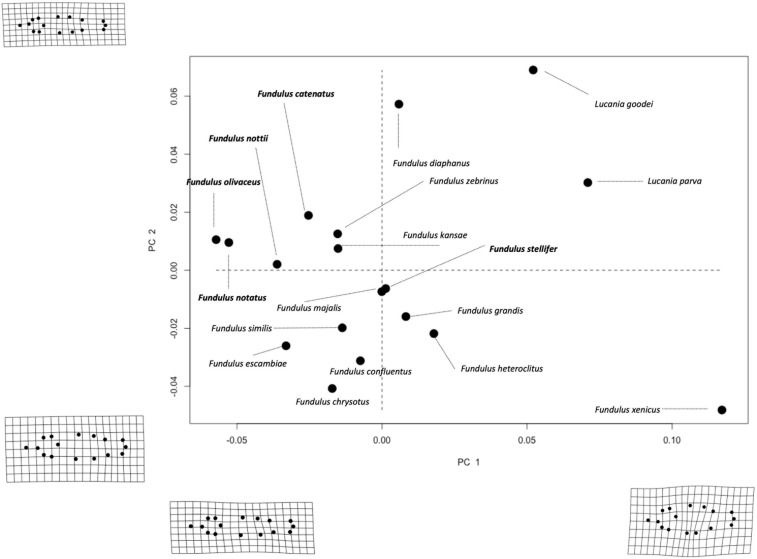
Bivariate PC plot of species averages separated according to salinity niche (bold = freshwater/brackish and plain text = saltwater species). Deformation grids (magnified 3×) associated with maximum and minimum values of each PC are shown on the corresponding axes. PC1 and 2 explain 52.3 and 23.2% of the variation in body shape among species, respectively.

A Generalized Procrustes superimposition was then used to remove isometric size, translation, and position (Rohlf and Slice, 1990) and to generate 32 Procrustes tangent coordinates (i.e., two coordinates per LM) (Adams and Nistri, 2010). The effect of location was removed by aligning the centroids (center of gravity) of all specimens to one common location in shape space (i.e., the origin). The effect of isometric size (scale) was removed from the analysis by dividing all specimens by their centroid size, which is the sum of the squared distances between each landmark and the centroid. Finally, we removed the effect of orientation from the shape analysis by rotating each landmark until the distance between specimens at that landmark were minimized (Mitteroecker and Gunz, 2009). A principal components analysis (PCA) was conducted to reduce dimensionality of the shape data (Lieberman et al., 2007). Procrustes distance, the difference in body shape at all of the landmarks between each specimen and the average specimen (Goswami and Polly, 2010), was also calculated. We used a combination of bivariate PC (principal component) plots and deformation grids to visualize shape differences among species and salinity niches/scopes. The first two PCs of shape variation, which explained 75.5% of the total variance (PC1 explained 52.3% of variance while PC2 explained 23.2; Figure 2), along with the Procrustes tangent coordinates were used in subsequent analyses.

### Phylogenetic Comparative Methods

#### Phylogeny Construction

**FIGURE 3:**
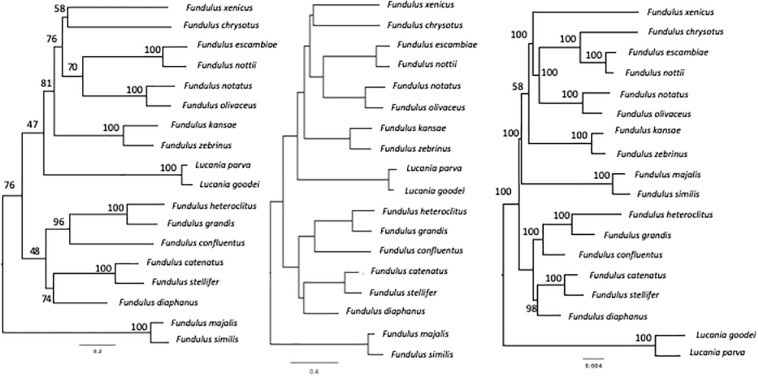
*Fundulus* phylogenies estimated from partitioned mixed-model maximum likelihood analysis of concatenated mitochondrial (cytb and CO1), nuclear (gylt, RAG1), and RNA-sequencing genetic data of Whitehead (2010) on the left, Ghedotti and Davis (2013) in the center, and Rodgers et al. (2018) on the right.

We used the RNA-sequencing data and evolutionary partitioning strategy of Rodgers et al. (2018) to re-create the most comprehensive phylogeny to date. In addition, using mitochondrial and nuclear DNA sequencing and the partitioning strategy of Whitehead (2010) and Ghedotti and Davis (2013), respectively, we re-created two additional phylogenies (Figure 3). These phylogenies were used as scaffolding to control for relevant phylogenetic influences while determining whether salinity niche and salinity scope may have driven variation in body shape, and FR at different stages of development in *Fundulus*. We conducted separate phylogenetic comparative analyses on each of the phylogenies. Additional phylogenetic comparative analyses were conducted using opercular epithelium surface area and gill arch length (see Supplementary Description A4 for description of variables).

#### Comparative Phylogenetic Approaches

Four different approaches were employed to investigate the relationship between salinity niche or salinity scope and morphology (body shape, and FR variation) among *Fundulus* species at three stages of development, and the methods are summarized in the sections below (see also Supplementary Description A3 and Supplementary Tables S2–S6). First, we used Adams (2014) distance-based-Phylogenetic Generalized Least Squares (PGLS; D-PGLS) on landmark data. This approach assumes trait evolution by multivariate BM and is suitable for testing for correlations between traits or between traits and environments while controlling for phylogenetic effects. We note, however, that phylogenetic effects are not necessarily equivalent to phylogenetic inertia (the real variable we need to control for). Thus we employed Hansen et al. (2008) univariate SLOUCH models as a second approach, using Procrustes distance as the response variable and environmentally determined, randomly evolving optima modeled on salinity niche and salinity scope. These models separate phylogenetic inertia (estimated as the rate of adaptation to new niche optima) from general phylogenetic effects, and only control for the former. They further assume that on macroevolutionary time scales, if a trait is under selection, most species’ traits should be at or near their optima (maintained by stabilizing selection). These optima can then be estimated by modeling the evolutionary trajectories of the traits across a phylogeny on various environmental variables that are hypothesized to affect the trait optima. By combining this modeling approach with Butler and King’s (2004) information criteria [Akaike’s Information Criterion corrected (AICc) for small sample sizes] model selection approach, the relative support for a number of competing adaptive and non-adaptive hypotheses can be quantified. Details of these models, and interpretations of the parameters they estimate are given in the Supplementary Material (see Supplementary Description A3 and Supplementary Table S2). Categorical salinity niche (freshwater/brackish or saltwater) and salinity scope (narrow or wide) variables were mapped onto phylogenetic trees with a Fitch parsimony algorithm, and minor ambiguities were dealt with by employing either delayed or accelerated transformation algorithms at ambiguous nodes (Fitch, 1971).

If Procrustes distance was significantly predicted by salinity niche or salinity scope, we used PC plots with species separated according to salinity niche or scope to determine what the shape variation associated with Procrustes distance actually represented. We did not directly compare among-species variance in PCs in response to salinity niche or scope because there are established evolutionary model biases in using PCs in a comparative phylogenetic framework (Uyeda et al., 2015). As a result, for our third approach, we used a combination of univariate and multivariate version of SLOUCH (Bartoszek et al., 2012) to evaluate whether among-species variation in the location of individual landmarks and landmark pairs in coordinate space was better explained by random processes (BM) or adaptive processes (OU) involving salinity niche optima. Given the number of parameters that need to be estimated for the fully multivariate approach (each parameter for the univariate model becomes a matrix of parameters with dimensions equal to the number of traits in the multivariate models), we could only estimate parameters with reasonable confidence for two landmark coordinates at a time. We furthermore had to assume that the rate of adaptation of the landmark pairs with respect to a given hypothesis was the same for all landmarks (see Supplementary Description A3, Figure 1, and Supplementary Tables S3–S6 for further justification of this approach, evidence that our assumption is reasonable, results from this comparative analysis, and optimal body shape estimates for each salinity classification as predicted by mvSLOUCH).

Fourth, we employed univariate SLOUCH models, using body shape (Procrustes distance), and FR as response variables and environmentally determined, randomly evolving optima modeled on salinity niche and salinity scope. For each trait, we ran six SLOUCH models. The first model assumed that each trait was evolving randomly under BM, void of any influence of salinity niche or salinity scope. The next five models assumed that each trait evolved under an OU adaptive process. The first of these assumed that each trait has evolved toward a single adaptive body shape or FR optima in response to one of the salinity variables. The next two models assumed each trait was evolving toward multiple salinity niches (freshwater/brackish or saltwater) mapped onto the phylogeny with both delayed or accelerated Fitch parsimony to deal with ambiguities. The next two models were the same as the previous two, except that traits were modeled toward salinity scope (“wide” or “narrow”) optima, instead of salinity niche optima. We used AICc (small sample size corrected Akaike Information Criteria) values to determine the most likely evolutionary model, and *p*-values to determine if salinity niche or salinity scope significantly predicted morphological variation. We included SL (or the length between the snout and beginning of the caudal fin) as a fixed allometric constant in all the models that included gill arch length and opercular epithelium surface area as a response variable because we were interested in determining if body size had a significant effect on these traits.

## Results

### D-PGLS and Body Shape Variation

**FIGURE 4:**
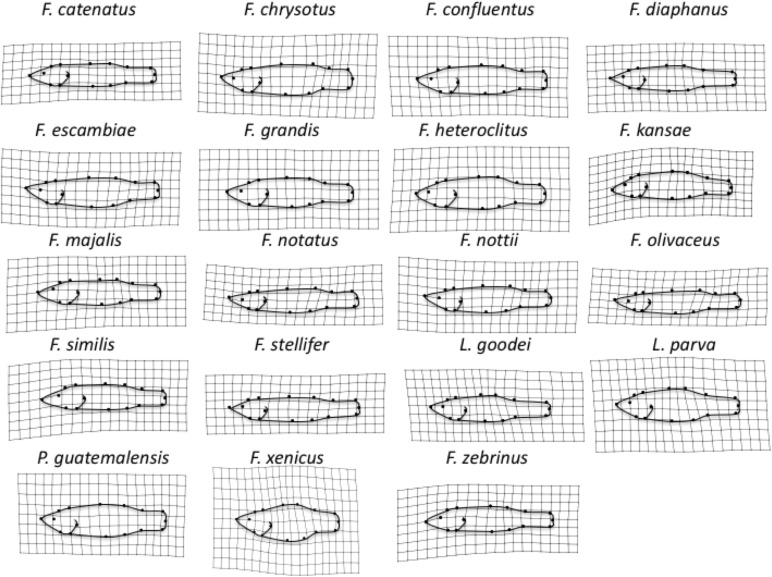
Average body shape across all three developmental stages for each of the 19 species, shown as a deformation of overall body shape for all specimens. Shape variation has been magnified 3× to enhance visualization.

Distance-based-Phylogenetic Generalized Least Squares demonstrated that variation among species in Procrustes Tangent Coordinates was correlated only with salinity niche in “old” fish (Supplementary Table S7). Neither salinity niche nor salinity scope was significantly correlated with species variation in Procrustes Tangent Coordinates in any other age group (Supplementary Table S7). PC1 and PC2 explained 75.5% of the variation in body shape among adult *Fundulus*. PC1 captured shape variation primarily along the dorsoventral axis, such that individuals with smaller PC1 scores had a more streamlined body shape and individuals with larger PC1 scores had a more robust body shape (Figure 2). PC2 captured shape variation along the dorsoventral axis, but much of this variation was concentrated in the caudal peduncle (i.e., the area between the dorsal/anal fins and the caudal fin). Saltwater species were much more robust and varied much more in body shape than freshwater/brackish species, which were streamlined by comparison (Figure 2). Shape variation averaged across all three developmental classes (“young,” “intermediate,” and “old”), for each species can be seen in Figure 4.

### Univariate SLOUCH Models for Procrustes Distance and FR

According to the univariate SLOUCH analysis, Procrustes distance did not vary systematically with either salinity niche or salinity scope, which was true regardless of inference method (i.e., *p*-values or maximum likelihood). With respect to FR, for all age groups, the univariate model that incorporated separate environmentally determined optima for salinity niche (freshwater/brackish vs. saltwater) fit our data significantly better than a model that had a single global FR optimum across all phylogenies (Table 1). Fish of all ages with saltwater physiologies possessed relatively deeper bodies than freshwater/brackish fish. For gill arch length and opercular epithelium surface area, across all age groups, the model that included body size as an isometric scaling variable, regardless of the environment (i.e., freshwater/brackish and saltwater) was consistently best across all phylogenies (Supplementary Table S8). In every scenario, there were negligible differences between models that reconstructed ancestral salinity states using accelerated Fitch parsimony and those that used decelerated Fitch parsimony so we present results based on the latter here (see Supplementary Figure S2).

**TABLE 1:** Models for FR (fineness ratio in mm) for Ghedotti and Davis (2013), Whitehead (2010), and Rodgers et al. (2018) trees.

		**Ghedotti**			**Whitehead**			**Rodgers**		
**FR (mm)**	**Model**	**Y**	**I**	**O**	**Y**	**I**	**O**	**Y**	**I**	**O**
	Salt	0	0	0	0	0	0	0	0	0
	Scope	2.97	4.31	4.29	3.3	2.27	4.13	2.93	2.58	1.49
	Global	5.11	4.73	3.55	5.53	2.55	3.16	5.05	3.61	1.94
	BM	6.65	7.17	6.78	6.76	4.55	6.54	6.16	3.53	1.93
Estimates − FR	Half life	0.09	0.07	0.06	0.14	0.13	0.11	0.02	1.18	0.5
	vy	0.28	0.26	0.26	0.27	0.27	0.27	0.27	1.47	0.49
	Saltwater	4.57	4.28	4.08	4.57	4.28	4.08	4.58	4.42	4.07
	Fresh/brackish	5.84	5.41	5.07	5.87	5.43	5.07	5.71	18.09	7.59
	r2 (%)	42.48	41.55	36.68	42.49	38.41	33.67	33.99	32.01	25.98
Support/±SEM − FR	Half life	0.00−0.21	0.00−0.79	0.00−0.59	0.00−2.34	0.00−1.01	0.00−1.01	0.00−0.21	0.00−∞	0.00−∞
	vy	0.21−0.56	0.21−0.56	0.21−0.50	0.18−0.58	0.18−0.58	0.18−0.58	0.18−0.58	0.60−∞	0.60−∞
	Saltwater	±0.16	±0.15	±0.15	±0.16	±0.15	±0.15	±0.14	±0.12	±0.13
	Fresh/brackish	±0.3	±0.27	±0.26	±0.31	±0.3	±0.28	±0.24	±4.67	±1.38

*For each of the phylogenies, the relative AICc value for each age group [“young”(Y), “intermediate” (I), and “old” (O)] is given. For each age group and each phylogeny, four models were competed against each other (BM = Brownian motion, Global = single optima OU model, Salt = two optima model for salt and brackish/freshwater fish, Scope = two optima model for fish with wide range salinity tolerance and narrow range salinity tolerance). The best model is listed first. In addition to AICc scores, point estimates and support values are given for each model (SEM = standard error of the mean, vy = stationary variance, and r2 = coefficient of determination).*

For the young age class, the saltwater fish optima for all phylogenies were close to the predicted FR optimum of 4.5, whereas freshwater/brackish fish were much longer and skinnier, with FR closer to 6 (Table 1, FR estimates). For the intermediate and old age classes, saltwater fish had a FR increasingly less than the predicted optimum of 4.5 (approaching four in larger fish), whereas the freshwater/brackish fish still had relatively higher FR, but closer to a value of five in two of phylogenies (and still large in the latter Rodgers Phylogeny, but we note much larger standard errors for these estimates as well, and suspect they represent estimation error based on the vastly greater non-ultrametric tree obtained for the Rodgers data set; see section “Discussion”).

### Multivariate SLOUCH Models for Landmark Pairs

**FIGURE 5:**
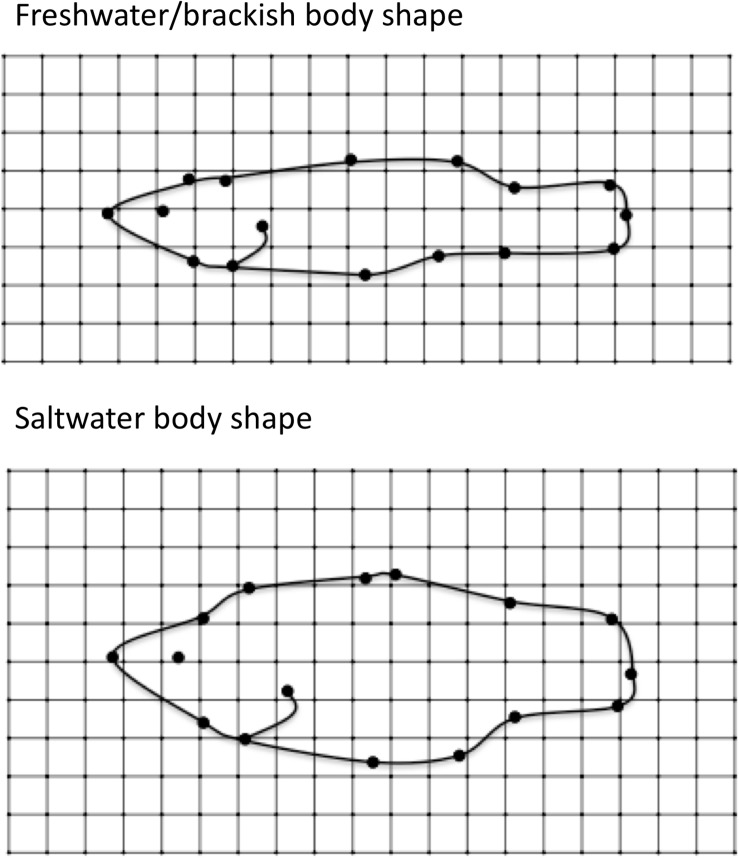
Optimal body shape as predicted by the adaptive bivariate OU predictor analysis. See Supplementary Material for a detailed description of OU predictor analysis. **(Top)** Optimal body shape of “freshwater” fish. **(Bottom)** Optimal body shape of saltwater fish.

When the individual landmark coordinates were modeled as adjacent (X) or opposite (Y) coordinate pairs in mvSLOUCH, the bivariate OU models that included Y3:Y11, Y6:Y8, X2:X3, X4:X5, X9:X10, X10:X11, and X11:X12 outperformed the multivariate BM models (Supplementary Table S4). OU bivariate predictor analysis for these coordinated pairs were then combined with average values for the other coordinates (which is the expectation if those other coordinates were evolving by BM), and used to plot the expected optimal shape of freshwater/brackish and saltwater species. Optimal fish shape in freshwater/brackish species was much more streamlined than the optimal shape of saltwater species (Figure 5).

## Discussion

Salinity niche predicts adaptive divergence in body shape and FR, while gill arch length and opercular epithelial surface area scale isometrically with body size in *Fundulus* at various stages in development. Regardless of which aspect of body shape was compared among species (i.e., Procrustes distance, landmark pairs, or FRs), and whether an adaptive or correlative macroevolutionary model was used, a clear pattern between historical salinity niche and body shape emerged. Relative to saltwater species, freshwater/brackish species possessed more streamlined body shapes. Species-level variation in gill arch length and opercular epithelium surface area, on the other hand, may be explained entirely by variation in body size. Other factors, including heterochrony (see Supplementary Description A4 and Supplementary Tables S9, S10 for a description and test of heterochrony in this *Fundulus* sample), do not seem to be primary drivers of variation among species in body shape.

Body shape may be related to salinity niche for a number of reasons. First, different flow rates, which may be predicted by salinity niche, can strongly influence body shape variation in fishes (Blob et al., 2008). In the case of *Fundulus*, if flow rate was a key selection pressure underlying body shape, then we would expect confidence intervals for optimal FRs in our best evolutionary model to include 4.5 because this is the FR at which resistance from drag would be minimized (Webb, 1975; Schaefer and Duvernell, 2011). However, the optimal FR confidence interval included 4.5 only for the saltwater salinity niche, a result that could be related to the highly variable flow regimes associated with saltwater habitats (e.g., estuaries, mangrove canals). In many saltwater habitats, high flow rates may arise from periodic and intense tidal fluctuation. Over time, this continual movement of water into and out of marine habitats may have selected for FRs closer to the hydrodynamic optimum in saltwater fish. Many of the freshwater/brackish species (*Fundulus catenatus*, *Fundulus notti*, *Fundulus notatus*, *Fundulus olivaceus*) included in this study do not occupy fast-flowing rivers and may thus may not experience flow rates strong enough to exert significant selection on body shape (Page and Burr, 2011).

Structural complexity may also covary with salinity niche and may be another factor influencing body shape evolution in *Fundulus*. Species inhabiting more complex habitats with obstructions to locomotion often have deep bodies and lower caudal fin aspect ratios, presumably as a result of strong, positive historical selection on unsteady swimming performance and maneuverability. Species inhabiting more open areas tend to have more streamlined body shapes, likely because of strong, positive historical selection on steady swimming (Langerhans and Reznick, 2010). Our results seem to support this trend considering that freshwater/brackish *Fundulus* species, which may spend more time feeding at the surface in open water (Carranza and Winn, 1954), were more streamlined than their saltwater counterparts, which may spend more time in structurally complex benthic and intertidal zones (Goto and Wallace, 2011).

Historical selection on niche-specific feeding behaviors may have driven the close association between body shape and salinity niche in *Fundulus*. This seems plausible considering that foraging behavior specific to different salinity niches appears to have influenced adaptive morphological divergence in other fish species (e.g., three-spined sticklebacks; Ravinet et al., 2014). For *Fundulus*, selection associated with feeding on fallen insects may have resulted in the evolution of an upturned mouth and flattened head that characterize some freshwater species including *F. notatus* and *F. olivaceus* (McKee and Parker, 1982). These traits would allow individuals to feed without the need to rotate their bodies anteriorly (Klinger et al., 1982), which would potentially make themselves vulnerable to benthic predators. Saltwater species specialized for feeding on suspended or sunken food items in the water column or substrate (Able et al., 2007), respectively, may have experienced relaxed selection on mouth position and body shape, and thus, evolved more varied phenotypes. For selection on feeding mode to have driven the close association between body shape and salinity niche, we would expect freshwater species not adapted for surface level feeding to exhibit body shapes more representative of their saline tolerant counterparts. This, however, does not appear to be the case. For instance, the freshwater fish *F. catenatus* spends most of the time feeding in the water column or substrate (Fisher, 1981) and still possesses a streamlined body.

Another factor that might have influenced the evolution of body shape in *Fundulus* is the abundance of gape-limited predators. In areas where predation is particularly high, fish often possess more robust bodies associated with lower FRs (Lostrom et al., 2015; Price et al., 2015). Perhaps saltwater *Fundulus* have lower FRs because predation pressure is higher in those environments relative to freshwater/brackish environments. However, as far as we know, the relative abundance of predators in freshwater/brackish vs. saltwater *Fundulus* communities has not been systematically examined. If predation was the primary driver of body shape variation in *Fundulus*, then we would expect significantly different body shapes in “young” fish in response to salinity niche because predation is especially high at this stage (Herrel and Gibb, 2006). This, however, was not observed.

Based on all of the evidence presented here, it appears that differential selection for osmoregulatory efficiency has been a major driver of body shape variation across ontogeny in *Fundulus*. Freshwater/brackish *Fundulus* are mostly hyperosmotic to their environment. Thus, barring any acid-base disturbance, freshwater fish must constantly combat influx of water across their skin and gills by producing large amounts of dilute urine and reabsorbing ions from well-developed glomeruli, an energetically costly process (Boeuf and Payan, 2001). Saltwater fish, on the other hand, must continue drinking water to avoid dehydration (Bielmyer et al., 2005). The reduced surface area associated with fusiform body shapes and high FRs seen in the freshwater/brackish *Fundulus* would be selectively advantageous compared to the wider body shape exhibited by the saltwater species because less water would diffuse passively into a fusiform body shape. Fish skin is permeable to water (Talbot et al., 1982) and, assuming that permeability is constant, the rate of diffusion between the environment and the fish should be directly proportional to concentration gradient x surface area (Fick’s Law). Thus, there should strong positive selection for bodies with less surface area in freshwater habitats, where the major challenge is water uptake.

The evolution of body shape variation in response to selection for osmoregulatory efficiency specifically, and not some other aspect of species’ salinity niche, becomes even more evident if we consider the natural history and observed variation in body shape of two *Fundulus* species in particular: *Fundulus kansae* and *Fundulus zebrinus*. These species occupy inland habitats similar to those occupied by saline intolerant species, except that the habitats have uncharacteristically high salinities (Griffith, 1974). Although *F. kansae* and *F. zebrinus* likely experience very different selection pressures (e.g., type and perhaps magnitude of predation, flow rate, etc.) than saltwater species, we found that they exhibit body shapes more characteristic of salinity tolerant species, implying that selection for osmoregulatory efficiency may eclipse other selection pressures associated with their salinity niche in terms of mediating morphological evolution.

Although few studies have examined macroevolution of body shape in response to variation in salinity, there is some evidence that salinity niche is an important predictor of morphological variation in a number of fish species. Varsamos et al. (2005) found that fish with higher salinity tolerance tended to develop gills with larger surface areas, and intestines and urinary systems that maximize water reuptake. Other studies have revealed links between body shape and salinity. Araújo and Monteiro (2012) found that *Poecilia vivipara* from areas with higher salinities tend to have deeper body profiles than *P. vivipara* found elsewhere, a trend that corresponds well with the present study. Although salinity seems to be at least partially responsible for this variation in body shape, Araújo and Monteiro (2012) could not separate variation in body shape due to selection for osmoregulation efficiency from alternative sources of selection.

Other studies that have investigated the relationship between salinity and body shape have reported conflicting results. For instance, Mazzarella et al. (2015) found that sticklebacks raised in marine conditions tended to develop a more streamlined body shape when compared to those raised in freshwater. However, Mazzarella et al. (2015) could not discriminate potential selection pressures that might have driven morphological variation among stickleback populations. It is possible that the trend uncovered by Mazzarella et al. (2015) reflects other selection pressures confounded with salinity that masked selection for osmoregulatory efficiency. Indeed, the body shape of ancestral marine populations is more robust than the derived freshwater populations (Aguirre and Bell, 2012), which seems to support the results in our study. Just as with *Fundulus*, future research is needed to understand the direct selection pressures associated with body shape evolution in sticklebacks (Walker, 1997; Shapiro et al., 2004). In addition, future studies should consider body shape variation associated with sex, considering that sexual dimorphism in body shape has been documented in *Fundulus* (Welsh et al., 2013).

Although salinity niche seems to predict variation in body shape and FR among different *Fundulus* species, neither salinity niche nor scope significantly predicted gill arch length. Instead, gill arch length scaled isometrically with body size. Importantly, however, gill arch length may not accurately reflect variation in other physiological mechanisms associated with gill osmoregulation in *Fundulus*. One species in particular, *F. heteroclitus*, has been particularly important model in identifying the physiological mechanisms associated with gill osmoregulation in teleosts (Whitehead et al., 2011). *F. heteroclitus* have high densities of mitochondrial rich cells (“chloride cells”) not only in their gill epithelium but also in their opercular epithelium, which allow them to more efficiently transport ions (Evans et al., 2005). Thus, the surface area of the opercular epithelium as well as gill arch length should vary among species in response to differences in historical selection imposed by salinity niche. In our study, however, neither the surface area of the opercular epithelium nor the length of the gill arch were significantly predicted by salinity niche at any age (see Supplementary Table S8 for results and Supplementary Description A4 for methods). In addition, different types of arginine vasotocin (AVT) and isotocin (IT) neuropeptide receptors may be present in the operculum of *F. heteroclitus*, which might aid in the effective transport of ions across the opercular epithelium (Martos-Sitcha et al., 2015). Aquaglyceroporin 3 (AQP3) expression in the pillar cells of the secondary lamellae also plays a role in water and ammonia exchange in *F. heteroclitus* (Jung et al., 2012). The degree to which these traits are found in other *Fundulus* species remains unclear. In addition, osmoregulatory capacity and other osmoregulatory mechanisms (e.g., Na/K-ATPase, cystic fibrosis transmembrane conductance regulator (CFTR), and Na-K-Cl cotransporter activity) are expected to vary across species as a function of salinity niche (Martos-Sitcha et al., 2015). Thus, future phylogenetic comparative studies of adaptations to salinity niche in this group would benefit by including these traits. However, other traits appear to reflect important osmoregulatory adaptations associated with evolutionary transitions between salinity niches in this family. For instance, Zimmer et al. (2019) found that saltwater-adapted killifish have more efficient Ca^2+^ uptake systems in their intestinal epithelia than freshwater killifish. Future work is needed to assess variation in these traits among *Fundulus* species and their relation to salinity niche to gain a better understanding of how osmoregulatory efficiency may have contributed to gill evolution in this group.

Because selection, and the variation available to selection, can vary at different stages of development (Ragland and Carter, 2004; Nilsson-Ortman et al., 2015), determining how the phenotype evolves depends heavily on our understanding of how selection culls (or maintains) variation throughout development. Despite this, few studies have examined the relationship between potential selection pressures and the phenotype outside of the adult stage (Herrel and Gibb, 2006; Gignac and O’Brien, 2016), while also comparing BM models to adaptive evolutionary models. Here, we provide strong evidence that selection imposed by historical salinity niches has driven the evolution of body shape at several stages of ontogeny in *Fundulus*. In doing so, we have highlighted that different salinity niches influence among-species variation in body shape in all stages of development.

## Data Availability Statement

The datasets generated for this study are available on request to the corresponding author.

## Author Contributions

JS collected the data, conducted the analysis, and led the writing of the manuscript. JP conducted the phylogenetic comparative analysis and contributed to the writing of the manuscript. PS conducted the phylogenetic reconstructions and contributed to the writing of the manuscript. RE contributed substantially to the writing of the manuscript and provided critical input into experimental design.

## Conflict of Interest

The authors declare that the research was conducted in the absence of any commercial or financial relationships that could be construed as a potential conflict of interest.
