# Supplementary figures and images for: Does Body Shape in Fundulus Adapt to Variation in Habitat Salinity?
Source: Front Physiol. 2019 Nov 15;10:1400. doi: 10.3389/fphys.2019.01400 (PMC6872640; doi:10.3389/fphys.2019.01400)

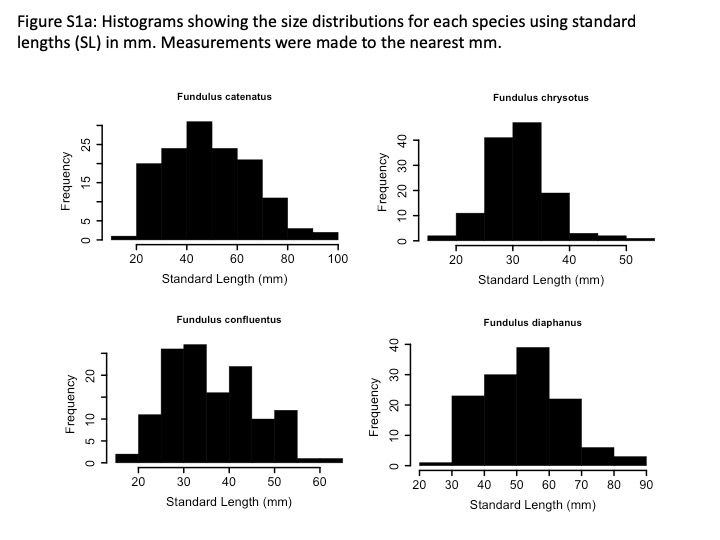

Supplement: Supplementary file 1 [file Image_1.JPEG]

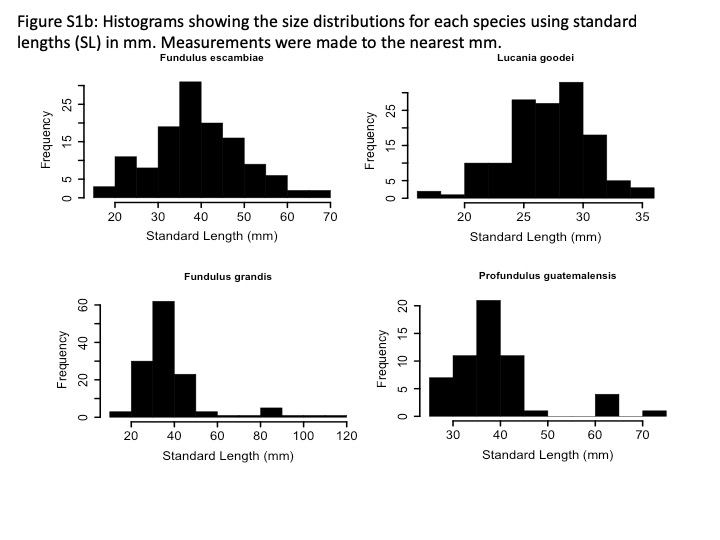

Supplement: Supplementary file 2 [file Image_2.JPEG]

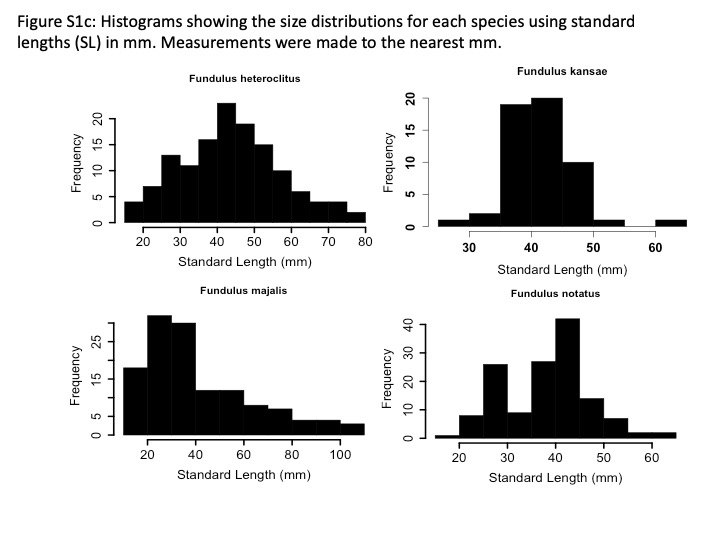

Supplement: Supplementary file 3 [file Image_3.JPEG]

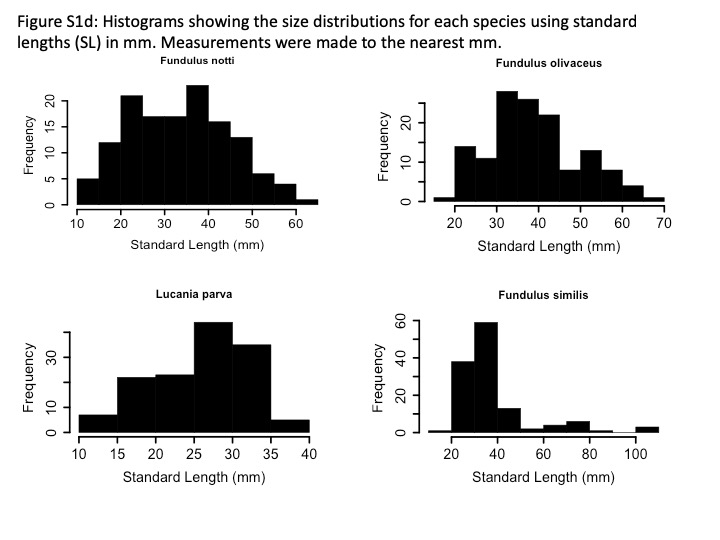

Supplement: Supplementary file 4 [file Image_4.JPEG]

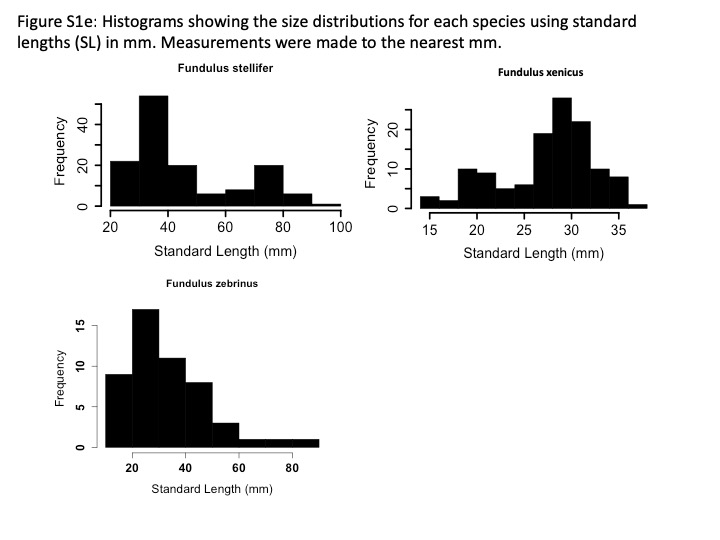

Supplement: Supplementary file 5 [file Image_5.JPEG]

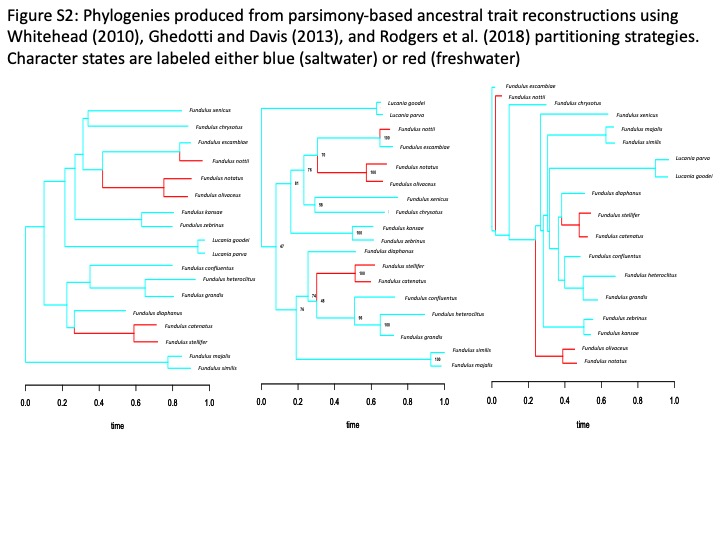

Supplement: Supplementary file 6 [file Image_6.JPEG]
