# Supplementary material for: Does Body Shape in Fundulus Adapt to Variation in Habitat Salinity?
Source: Front Physiol. 2019 Nov 15;10:1400. doi: 10.3389/fphys.2019.01400 (PMC6872640; doi:10.3389/fphys.2019.01400)
Supplement: Supplementary file 7 [file Table_1.docx]

**Table S1:** General distribution, salinity classification (S=saltwater (>25 ppt max), F=freshwater (<25 ppt max), Scope (W=wide (>1 ppt), or N=narrow (<1 ppt), and Museum Catalogue numbers of select *Fundulus* species.

| Species | Class | Scope | N | Salinity reference(s) | Museum Catalogue numbers | Collection location(s) |
| --- | --- | --- | --- | --- | --- | --- |
| *Fundulus catenatus* | F | N | 137 | Ghedotti & Davis (2013) | UA 3202; UA 4003; UA 4774.13; UA 4813.11;  UA 4861.14; UA 8416.1;  UA 10342.13; UA 11156.1 | Lower TN, North Ozarks, Ouachita, South Ozarks |
| *Fundulus chrysotus* | S | N | 126 | Ghedotti & Davis (2013) | UA 3176;  UA 2650 | Alachua Co., Choctawhatchee |
| *Fundulus confluentus* | S | W | 128 | Ghedotti & Davis (2013) | UA 4588.07; UA 3017;  UA 1666; UA 3722;  UA 1602; UA 4019 | W. Atlantic, Escatawpa, Mississippi Sound, Mobile Bay, Palm Beach, Satilla |
| *Fundulus diaphanus* | S | W | 124 | Ghedotti & Davis (2013) | UA 12060.02;  UA 13603.06;  UA 2723 | Fox, Hudson, Lake Huron |
| *Fundulus escambiae* | S | N | 127 | Ghedotti & Davis (2013) | UA 5821.03; UA 11085.1; UA 8832.02; UA 417; UA 7872.01;  UA 10504.06; UA 6769.08; UA 6438.02; UA 9995.08; UA 12603.04;  UA 12593.03; UA 9187.09; UA 10854.01; UA 5821.03 | Blackwater, Choctawhatchee, Escambia, Mobile Bay, Ochlochonee, Perdido, Suwannee, Yellow |
| *Lucania goodei* | S | W | 137 | Ghedotti & Davis (2013) | UA 8932.01; UA 2647 | Gulf of Mexico, Tampa Bay |
| *Fundulus grandis* | S | W | 131 | Ghedotti & Davis (2013) | UA 4509.09; UA 4665.04 | Gulf of Mexico, W. Atlantic |
| *Profundulus guatemalensis* | F | NA | 56 | NA | UA 9552.02 | Lago de Amatitlan |
| *Fundulus heteroclitus* | S | W | 134 | Ghedotti & Davis (2013) | UA 1; UA 2657; UA 4590.1; UA 872; UA 177 | Atlantic-FL, Atlantic-NY, Eastman Creek-NC |
| *Fundulus kansae* | S* | W | 54 | Ghedotti & Davis (2013) | N/A | UNKNOWN |
| *Fundulus majalis* | S | W | 130 | Ghedotti & Davis (2013) | UA 1440; UA 4590.11; UA 1600; UA 4591.08 | Atlantic-FL, Atlantic-NY, St. Simmons Island |
| *Fundulus notatus* | F | N | 138 | Ghedotti & Davis (2013) | UA 3163; UA 2292; UA 1890.06: UA 2991.13;UA 8634.03 | Lower Mississippi, Lower TN, Ohio, Upper Tombigbee |
| *Fundulus nottiii* | F | N | 135 | Ghedotti & Davis (2013) | UA 4007; UA 1733; UA 1088; UA 437; UA 340; UA 6371.01 | Escatawpa, Lower Tombigbee, Mobile, St. Louis Bay |
| *Fundulus olivaceus* | F | N | 136 | Ghedotti & Davis (2013) | UA 878; UA 1738; UA 7108.06; UA 3821; UA 3843; UA 7190.19;  UA 3821; UA 3843; UA 7190.19; UA 3588 | Black Warrior, Cahaba, St. Louis Bay, Upper Tombigbee |
| *Lucania parva* | S | W | 136 | Ghedotti & Davis (2013) | UA 2386; UA 872; UA 4019; UA 2825; UA 1291 | Atlantic, Blackwater, Escatawpa, Gulf of Mexico, |
| *Fundulus similis* | S | W | 127 | Ghedotti & Davis (2013) | UA 4151.07; UA 4067; UA 4046 | Gulf of Mexico, Mississippi Sound, Mobile Bay |
| *Fundulus stellifer* | F | N | 137 | Ghedotti & Davis (2013) | UA 15028.02; UA 15035.02; UA 15038.02; UA 15030.02;  UA 15036.02 | Coosa |
| *Fundulus xenicus* | S | W | 123 | Ghedotti & Davis (2013) | UA 3017; UA 4068; UA 4694.06; UA 4076; UA 732 | Cat island, Dauphin Island, Mobile, Pascagoula Bay, Perdido Bay, |
| *Fundulus zebrinus* | S* | W | 51 | Ghedotti & Davis (2013) | UA 1020.06; UA 8597.02; UA 11238.11; UA 12293.04; UA 13511.01  UA 13651.08 | Cheyenne River, Green River, Pecos River, Republican River, South Platte River, Wild Horse Creek |

* Although these species are classified as marine, they occur inland in isolated pools with high salinity levels.

**Table S2** Parameters estimated by SLOUCH and mvSLOUCH and how they are interpreted.

| **Parameter** | **Statistical support** | **Interpretation** |
| --- | --- | --- |
| α | Joint support region with σ^2^_Y_ (see below). The support region of the set of values for both α and σ^2^_Y_ values that result in a likelihood support within two units of the maximum likelihood value | “Rate of adaptation” – the larger the value, the faster traits switch from one trajectory to another. Thus, as environmentally determined optima change, alpha determines how fast the traits can track the changes. The parameter enters both into the mean structure of the model, as well as the residual variance structure where it modulates the influence of phylogenetic relatedness on residual covariances. It does so on an exponential scale however, so it can be difficult to interpret. For this reason, Hansen & colleagues (1997, 2005, 2008, 2012) suggest using a transformation of this parameter: ln(2)/α, or the phylogenetic half-life, that is on the same linear scale as the branch lengths (below for interpretation). |
| β_0_ (optimal) | Standard errors | Intercept for the univariate optimal regression (fixed or random, see below). |
| βi (optimal) | Standard errors | Slope (or slopes) for a univariate single or multiple regression that relate environmental variables to the “optima” for traits by predicting the expected “optimal” relationship for a regression of traits on environmental variables, if there were no influence of phylogenetic inertia (or if selection were to continue the same trajectory indefinitely). It can be fixed or random, depending on how environmental variables are modeled. If random, environmental variables themselves are assumed to evolve as a Brownian motion process. |
| β_0_ (evolutionary) | Standard errors | “Observed” regression intercept for the regression of a trait on environmental variables, corrected for phylogenetic effects (note that phylogenetic effects are not the same as inertia; the former are phylogenetically correlated residuals, not modulated by the alpha parameter). |
| β_i_ (evolutionary) | Standard errors | “Observed” regression slope for the regression of a trait on environmental variables, corrected for phylogenetic effects (note that phylogenetic effects are not the same as inertia; the former are phylogenetically correlated residuals, not modulated by the alpha parameter). |
| σ^2^_Y_ | See α | Stationary variance of the Ornstein-Uhlenbeck process (if environmental variables are assumed to be fixed as in a fixed factor ANOVA or ANCOVA) or the joint Ornstein-Uhlenbeck / Brownian motion variance if the environment is modeled as a randomly evolving Brownian Motion. This parameter captures variance in trait values through time generated by random processes (for example, small unmeasured selective forces, random genetic drift, environmental influences etc.) as with α, it is often more meaningful to scale this parameter in some ways; (σ^2^_Y_/2α), for example, allows us to capture a stationary variance, V_y_. |
| σ^2^_x_ | Standard errors | Variance of the Brownian motion process, it determines the magnitude of change in the predictor variables (for multiple predictors, this parameter is a linear combination of their individual variances). |
| t_1/2_ | See α | “Phylogenetic half-life” – is a measure of phylogenetic inertia. Like elemental decay half-lives, it is interpreted as the time it takes for half the influence of the ancestral trait value to disappear from current trait values adapting to changing fitness peaks. It is interpreted on the same scale as the branch length units. For example, if a phylogenetic tree is one million years in height, and the half-life estimate is 0.5, this means it takes half a million years on average for the ancestral trait influence to disappear from current trait values (strong phylogenetic inertia). |
| θ | Standard errors | Mean of the Brownian motion process describing random evolution of continuous predictor variables. |

**Table S3:** Univariate SLOUCH results for landmark positions modeled on Salinity Scope, Salinity Max and Salinity Average. Best models as determined by AICc are highlighted in yellow. LM = landmark; BM = Brownian Motion model; t_1/2_ = phylogenetic half-life.

|  | **BM** |  | **Scope** | | |  | **Max** | | |  | **Avg** | | |
| --- | --- | --- | --- | --- | --- | --- | --- | --- | --- | --- | --- | --- | --- |
| **LM** | **AICc** |  | **AICc** | **R^2^ (%)** | **t_1/2_** |  | **AICc** | **R^2^ (%)** | **t_1/2_** |  | **AICc** | **R^2^ (%)** | **t_1/2_** |
| X1 | ***-107.56*** |  | -106.34 | 9.67 | 0.4 |  | -109.09 | 22.69 | 0.4 |  | -104.96 | 3.63 | 0.4 |
| X2 | 124.61 |  | ***-130.95*** | 63.04 | 1.9 |  | -125.27 | 0.03 | 1.6 |  | -125.42 | 1.09 | 1.6 |
| X3 | -121.98 |  | *-****137.91*** | 53.18 | 0.2 |  | -123.30 | 10.22 | 1.3 |  | -123.17 | 10.69 | 1.3 |
| X4 | *-****105.57*** |  | -99.54 | 0.15 | 0.4 |  | -107.24 | 29.48 | 0.4 |  | -101.37 | 8.85 | 1.8 |
| X5 | ***-79.29*** |  | -79.88 | 9.56 | 0.3 |  | -79.39 | 52.68 | 1.9 |  | -**79.56** | 14.48 | 1.2 |
| X6 | -84.00 |  | ***-99.85*** | 65.46 | 2.2 |  | -**98.33** | 53.43 | 1.8 |  | -88.32 | 0.82 | 2.3 |
| X7 | -104.40 |  | -105.21 | 0.89 | 0.2 |  | ***109.36*** | 11.42 | 3.0 |  | -109.11 | 0.00 | 2.1 |
| X8 | -100.52 |  | **-106.99** | 1.42 | 0.1 |  | *-****107.43*** | 4.35 | 0.1 |  | -**106.80** | 0.08 | 0.1 |
| X9 | -111.48 |  | -109.63 | 2.66 | 0.5 |  | *-****117.47*** | 10.70 | 3.0 |  | -109.23 | 0.55 | 0.4 |
| X10 | -101.11 |  | ***-114.40*** | 0.11 | 0.0 |  | **-114.36** | 4.11 | 0.0 |  | **-114.30** | 2.70 | 0.0 |
| X11 | -119.38 |  | ***122.37*** | 14.10 | 1.1 |  | -119.10 | 5.76 | 1.0 |  | -118.49 | 2.21 | 1.0 |
| X12 | -111.20 |  | ***121.00*** | 13.16 | 0.5 |  | **-119.79** | 49.46 | 0.7 |  | -110.45 | 0.00 | 2.1 |
| X13 | *-****117.30*** |  | -117.11 | 5.28 | 0.8 |  | -118.79 | 22.47 | 2.5 |  | 116.44 | 1.03 | 0.7 |
| X14 | -171.10 |  | *-****178.04*** | 19.97 | 0.1 |  | -172.50 | 34.03 | 2.1 |  | 170.94 | 0.39 | 0.1 |
| X15 | -199.90 |  | ***212.72*** | 68.63 | 1.7 |  | -206.81 | 27.74 | 1.4 |  | 198.95 | 7.32 | 0.9 |
| X16 | -105.20 |  | ***-114.00*** | 67.12 | 1.7 |  | -106.95 | 15.00 | 0.3 |  | -103.84 | 0.30 | 0.3 |
| Y1 | -106.44 |  | ***-110.92*** | 44.09 | 3.0 |  | -106.05 | 16.00 | 0.3 |  | 106.00 | 15.00 | 0.4 |
| Y2 | 120.44 |  | *-****133.67*** | 54.87 | 0.1 |  | -121.74 | 15.00 | 1.2 |  | -119.32 | 0.40 | 1.0 |
| Y3 | -108.23 |  | ***-115.67*** | 55.29 | 0.0 |  | -112.26 | 30.27 | 0.1 |  | -109.33 | 4.34 | 0.2 |
| Y4 | -85.73 |  | ***-101.68*** | 42.07 | 0.1 |  | 91.75 | 12.95 | 0.1 |  | -91.65 | 12.16 | 0.1 |
| Y5 | -91.45 |  | ***-97.42*** | 36.41 | 0.1 |  | -89.03 | 2.69 | 2.3 |  | -88.99 | 4.25 | 2.2 |
| Y6 | -109.80 |  | *-****114.68*** | 26.44 | 0.1 |  | -108.27 | 2.59 | 0.4 |  | -108.06 | 1.63 | 0.3 |
| Y7 | ***-126.00*** |  | **-125.48** | 15.33 | 1.6 |  | -123.55 | 0.02 | 1.5 |  | **-124.82** | 9.80 | 1.6 |
| Y8 | -137.89 |  | -137.10 | 12.73 | 0.1 |  | **-144.17** | 36.76 | 0.1 |  | *-****145.50*** | 38.38 | 0.2 |
| Y9 | -122.90 |  | ***-127.24*** | 44.49 | 2.5 |  | -122.47 | 14.06 | 1.3 |  | -120.19 | 0.60 | 1.2 |
| Y10 | -118.00 |  | ***-121.60*** | 21.01 | 1.0 |  | -116.61 | 0.50 | 0.9 |  | 116.57 | 0.26 | 0.9 |
| Y11 | -95.90 |  | ***-99.21*** | 27.63 | 0.2 |  | -92.93 | 0.37 | 3.0 |  | -92.93 | 0.69 | 3.0 |
| Y12 | -92.26 |  | ***-98.01*** | 36.90 | 0.2 |  | -89.68 | 2.30 | 2.4 |  | -89.38 | 0.26 | 2.4 |
| Y13 | -108.37 |  | *-114.30* | 39.70 | 0.8 |  | -108.31 | 8.63 | 0.3 |  | -106.88 | 0.19 | 0.3 |
| Y14 | -121.57 |  | *-129.53* | 40.79 | 0.1 |  | -125.29 | 43.61 | 2.2 |  | -120.20 | 1.07 | 1.0 |
| Y15 | -133.06 |  | *-142.41* | 28.28 | 0.1 |  | -136.21 | 7.50 | 0.1 |  | -135.12 | 3.79 | 0.1 |
| Y16 | *-125.13* |  | 122.56 | 4.20 | 1.5 |  | -122.12 | 1.17 | 1.4 |  | -122.00 | 0.44 | 1.4 |

**Table S4:** Multivariate landmark pairs analyzed by mvSLOUCH. The AICc values compare a model where the traits coevolve as if by Brownian motion compared to a model where Salinity Scope affects the optima for each of the traits (and a given traits optimum, affects the other traits optimum as well). Best models are italicized in yellow. BM = Brownian Motion model; mvOU = multivariate OU model; MV t½ = the joint phylogenetic half life for a pair of landmarks; LM1 t½ and LM2 t½ = the phylogenetic half-life for the first (LM1) and second (LM2) landmark listed for a pair of landmarks as estimated by univariate SLOUCH

| **Landmark pair** | **BM** | **mvOU** | **R^2^ (%)** | **MV _t½_** | **LM1 _t½_** | **LM2 _t½_** |
| --- | --- | --- | --- | --- | --- | --- |
| Y3-Y11 | -51.77 | *-54.47* | 31.00% | 0.31 (0.15-∞) | 0.00 (0.00-0.24) | 0.20 (0.00-1.60) |
| Y4-Y10 | *-74.17* | -62.12 |  |  |  |  |
| Y13-Y14 | *-92.24* | -88.00 |  |  |  |  |
| Y5-Y9 | *-71.39* | -60.00 |  |  |  |  |
| Y6-Y8 | -81.67 | *-88.08* | 29.00% | 0.28 (0.14-∞) | 0.10 (0.00-1.20) | 0.10 (0.00-0.60) |
| Y2-Y12 | *-67.82* | -57.55 |  |  |  |  |
| X8-X9 | *-65.99* | -53.00 |  |  |  |  |
| X5-X6 | *-18.30* | 15.47 |  |  |  |  |
| X3-X4 | *71.06* | -59.50 |  |  |  |  |
| X2-X3 | -103.55 | *-104.59* | 28.28 % | 0.18 (0.07-∞) | 1.87 (1.55-2.40) | 0.18 (0.00-1.01) |
| X1-X2 | *-67.05* | -66.14 |  |  |  |  |
| X1-X12 | *-63.72* | -56.89 |  |  |  |  |
| X11-X12 | -64.22 | *-71.42* | 12.85 % | 1.13 (0.51-∞) | 1.10 (0.50-1.20) | 0.50 (0.41-0.81) |
| X10-X11 | -63.08 | *-77.93* | 8.034% | 0.069 (0.0-0.50) | 0.00 (0.0-0.12) | 1.10 (0.0-∞) |
| X9-X10 | -55.47 | *-62.45* | 2.25% | 0.11 (0.05-∞) | 0.50 (0.00-1.20) | 0.00 (0.0-0.22) |
| X4-X5 | -15.66 | *-18.39* | 18.02% | 0.30 (0.15-∞) | 0.40 (0.10-1.30) | 0.30 (0.11-0.75) |

**Table S5:** Univariate SLOUCH results for individual landmark coordinates. AICc values for Brownian motion and adaptive hypotheses (Ornstein-Uhlenbeck, OU; salinity scope, maximum salinity tolerance, and average salinity tolerance), coefficients of determination (R^2^), and phylogenetic half-lives (h) are given. The best model(s) is identified in bold and with an asterisk.

| **Landmark** | **BM** | **OU-Scope** |  |  | **OU-Max** |  |  | **OU-Avg** |  |  |
| --- | --- | --- | --- | --- | --- | --- | --- | --- | --- | --- |
|  | **AICc** | **AICc** | **R^2^ (%)** | **h** | **AICc** | **R^2^ (%)** | **h** | **AICc** | **R^2^ (%)** | **h** |
| X1 | **-107.56*** | -106.34 | 9.67 | 0.4 | **-109.09*** | 22.69 | 0.40 | -104.96 | 3.63 | 0.40 |
| X2 | 124.61 | **-130.95*** | 63.04 | 1.9 | -125.27 | 0.03 | 1.60 | -125.42 | 1.089 | 1.60 |
| X3 | -121.98 | -**137.91*** | 53.18 | 0.2 | -123.30 | 10.22 | 1.30 | -123.17 | 10.69 | 1.30 |
| X4 | -**105.57*** | -99.54 | 0.15 | 0.4 | **-107.24*** | 29.48 | 0.40 | -101.37 | 8.85 | 1.80 |
| X5 | **-79.29*** | -**79.88*** | 9.56 | 0.3 | **-79.39*** | 52.68 | 1.90 | -**79.56*** | 14.48 | 1.20 |
| X6 | -84.00 | **-99.85*** | 65.46 | 2.2 | -**98.33*** | 53.43 | 1.80 | -88.32 | 0.82 | 2.30 |
| X7 | -104.4 | -105.21 | 0.89 | 0.2 | **109.36*** | 11.42 | 3.00 | -109.11 | 0.00 | 2.10 |
| X8 | -100.52 | **-106.99*** | 1.42 | 0.1 | -**107.43*** | 4.35 | 0.10 | -**106.80*** | 0.08 | 0.10 |
| X9 | -111.48 | -109.63 | 2.66 | 0.5 | -**117.47*** | 10.70 | 3.00 | -109.23 | 0.55 | 0.40 |
| X10 | -101.11 | **-114.36*** | 0.11 | 0.0 | **-114.40*** | 4.11 | 0.00 | **-114.30*** | 2.70 | 0.00 |
| X11 | -119.38 | **122.37*** | 14.10 | 1.1 | -119.10 | 5.76 | 1.00 | -118.49 | 2.21 | 1.00 |
| X12 | -111.20 | **121.00*** | 13.16 | 0.5 | **-119.79*** | 49.46 | 0.70 | -110.45 | 0.00 | 2.10 |
| X13 | -**117.30*** | -**117.11** | 5.28 | 0.8 | **-118.79*** | 22.47 | 2.50 | 116.44 | 1.03 | 0.70 |
| X14 | -171.10 | -**178.04*** | 19.97 | 0.1 | -172.50 | 34.03 | 2.10 | 170.94 | 0.39 | 0.10 |
| X15 | -199.90 | **212.72*** | 68.63 | 1.7 | -206.81 | 27.74 | 1.40 | 198.95 | 7.32 | 0.90 |
| X16 | -105.20 | **-114.00*** | 67.12 | 1.7 | -106.95 | 15.00 | 0.30 | -103.84 | 0.30 | 0.30 |
| Y1 | -106.44 | **-110.92*** | 44.09 | 3 | -106.05 | 16.00 | 0.30 | 106.00 | 15.0 | 0.40 |
| Y2 | 120.44 | -**133.67*** | 54.87 | 0.1 | -121.74 | 15.00 | 1.20 | -119.32 | 0.4 | 1.00 |
| Y3 | -108.23 | **-115.67*** | 55.29 | 0 | -112.26 | 30.27 | 0.10 | -109.33 | 4.34 | 0.20 |
| Y4 | -85.73 | **-101.68*** | 42.07 | 0.1 | 91.75 | 12.95 | 0.10 | -91.65 | 12.16 | 0.10 |
| Y5 | -91.45 | **-97.42*** | 36.41 | 0.1 | -89.03 | 2.69 | 2.30 | -88.99 | 4.25 | 2.20 |
| Y6 | -109.80 | -**114.68*** | 26.44 | 0.1 | -108.27 | 2.59 | 0.40 | -108.06 | 1.63 | 0.30 |
| Y7 | **-126.00*** | **-125.48*** | 15.33 | 1.6 | -123.55 | 0.02 | 1.50 | **-124.82*** | 9.80 | 1.60 |
| Y8 | -137.89 | -137.10 | 12.73 | 0.1 | **-144.17*** | 36.76 | 0.10 | -**145.50*** | 38.38 | 0.20 |
| Y9 | -122.90 | **-127.24*** | 44.49 | 2.5 | -122.47 | 14.06 | 1.30 | -120.19 | 0.60 | 1.20 |
| Y10 | -118.00 | **-121.60*** | 21.01 | 1 | -116.61 | 0.50 | 0.90 | 116.57 | 0.26 | 0.90 |
| Y11 | -95.90 | **-99.21*** | 27.63 | 0.2 | -92.93 | 0.37 | 3.00 | -92.93 | 0.69 | 3.00 |
| Y12 | -92.26 | **-98.01*** | 36.90 | 0.2 | -89.68 | 2.3 | 2.40 | -89.38 | 0.26 | 2.40 |
| Y13 | -108.37 | **-114.30*** | 39.70 | 0.8 | -108.31 | 8.63 | 0.30 | -106.88 | 0.19 | 0.30 |
| Y14 | -121.57 | **-129.53*** | 40.79 | 0.1 | -125.29 | 43.61 | 2.20 | -120.20 | 1.07 | 1.00 |
| Y15 | -133.06 | **-142.41*** | 28.28 | 0.1 | -136.21 | 7.5 | 0.10 | -135.12 | 3.787 | 0.10 |
| Y16 | -125.13 | 122.56 | 4.199 | 1.5 | -122.12 | 1.17 | 1.40 | -122.00 | 0.44 | 1.40 |

**Table S6:** Optimal slope and intercept regression estimates used to calculate predicted landmark (LM) positions if adaptation to salinity scope were instantaneous.

| **LM** | **Bi (optimal)** | **B0 (optimal)** | **Predicted Min Value (0.025)** | **Predicted Max Value (84.5)** |
| --- | --- | --- | --- | --- |
| Y3 | -0.000707824 | -0.05226746 | -0.052285156 | -0.112078554 |
| Y11 | 0.00083712 | 0.05590499 | 0.055925918 | 0.126641638 |
| Y6 | -0.000571534 | -0.0424443 | -0.042458588 | -0.090738948 |
| Y8 | 0.000159845 | -0.00300487 | -0.003000874 | 0.01050199 |
| X2 | -0.000157121 | 0.2548977 | 0.254893772 | 0.24162095 |
| X3 | -0.000304082 | 0.2020822 | 0.202074598 | 0.176387305 |
| X10 | -2.43E-05 | -0.2002238 | -0.200224408 | -0.202279459 |
| X11 | -1.42E-04 | -0.1111913 | -0.111194844 | -0.123169234 |
| X10 | -3.25E-05 | -0.200107 | -0.200107814 | -0.202857045 |
| X9 | 1.02E-04 | -0.3571836 | -0.357181054 | -0.34857654 |
| X4 | -0.00013154 | 0.02155593 | 0.021552641 | 0.010440783 |
| X5 | 0.001177704 | -0.13187394 | -0.131844497 | -0.032357977 |

**Table S7**: Results from D-PGLS for salinity niche or salinity scope of Procrustes Tangent Coordinates for the three age groups.

| Age groups | Predictor | F_(Num DF, Den DF)_, p-value |
| --- | --- | --- |
| Young | Niche | F_(1, 16)_=0.67, p=0.42 |
|  | Scope | F_(1, 16)_=0.064, p=0.80 |
| Intermediate | Niche | F_(1, 16)_=0.14, p=0.71 |
|  | Scope | F_(1, 16)_=0.85, p=0.37 |
| Old | Niche | F_(1, 16)_=0.30, p=0.59 |
|  | Scope | **F_(1, 16)_=5.70, p=0.029*** |

**Table S8:** Models for Gill arch length (mm) for three *Fundulus* phylogenies, including Ghedotti and Davis (2013), Whitehead (2010), and Rodgers et al. (2018). For each phylogeny, we included the relative AICc for each age group (‘young (Y), ‘intermediate’ (I) and ‘old” (O)). Four models were competed for each phylogeny. These models included a Brownian motion model (BM), an Ornstein Ulhenbeck (OU) model with a single optimum (Global), an OU model with two separate optima for each salinity regime (Salt), and an OU model with two separate optima for salinity tolerance (i.e. narrow vs wide salinity tolerance). The most likely model is given first. We also include point estimates and support values for each model. These included standard error of the mean (SEM), stationary variance (vy), intercept of regression (Bo), regression intercept (Bi), and coefficient of determination (r2). We did not include the results for the models involving the response of opercular surface area (mm^2^) to salinity niche because they were qualitatively identical to those associated with gill arch length.

|  |  | **Ghedotti** | | | **Whitehead** | | | **Rodgers** | | |
| --- | --- | --- | --- | --- | --- | --- | --- | --- | --- | --- |
| **Gill (mm)** | **Model** | **Y** | **I** | **O** | **Y** | **I** | **O** | **Y** | **I** | **O** |
|  | **BodyL** | ***0*** | ***0*** | ***0*** | ***0*** | ***0*** | ***0*** | ***0*** | ***0*** | ***0*** |
|  | **Global** | 16.77 | 21.41 | 57.95 | 11.31 | 16.39 | 26.95 | 12.16 | 18.69 | 24.4 |
|  | **Scope** | 18.18 | 24.22 | 60.06 | 12.72 | 19.25 | 29.13 | 13.57 | 21.17 | 26.81 |
|  | **Salt** | 19.72 | 24.74 | 61.3 | 14.26 | 19.73 | 30.28 | 15.11 | 22.01 | 27.72 |
|  | **BM** | 27.58 | 34.71 | 62.8 | 20.24 | 27.43 | 29.25 | 20.38 | 22.41 | 25.43 |
|  |  |  |  |  |  |  |  |  |  |  |
| Estimates - Gill | **half life** | 0.04 | 0.15 | 0.16 | 0 | 0.18 | 0.23 | 0 | 0.02 | 0 |
|  | **vy** | 0.07 | 0.19 | 0.2 | 0.07 | 0.19 | 0.2 | 0.07 | 0.18 | 0.2 |
|  | **Bo** | 0.32 | -0.092 | -0.13 | 0.34 | -0.001 | -0.07 | 0.35 | 0.3 | -0.05 |
|  | **Bi** | 0.084 | 0.01 | 0.01 | 0.083 | 0.097 | 0.01 | 0.083 | 0.087 | 0.097 |
|  | **r2 (%)** | 67.63 | 76.64 | 81.83 | 67.17 | 74.87 | 80.83 | 67.17 | 69.07 | 77.02 |
|  |  |  |  |  |  |  |  |  |  |  |
| Support/ ± SEM - Gill | **half life** | 0.00 - 0.38 | 0.00 - 0.38 | 0.00 - 0.38 | 0.00 - 0.36 | 0.00 - 0.36 | 0.00 - 0.36 | 0.00 - 0.37 | 0.00 - 0.37 | 0.00 - 0.37 |
|  | **vy** | 0.02-0.16 | 0.02-0.16 | 0.02-0.16 | 0.02-0.16 | 0.02-0.16 | 0.02-0.16 | 0.03 - 0.017 | 0.03 - 0.017 | 0.03 - 0.017 |
|  | **Bo** | ± 0.52 | ± 0.46 | ± 0.36 | ± 0.51 | ± 0.47 | ± 0.37 | ± 0.51 | ± 0.49 | ± 0.36 |
|  | **Bi** | ± 0.02 | ± 0.013 | ± 0.01 | ± 0.03 | ± 0.01 | ± 0.01 | ± 0.083 | ± 0.087 | ± 0.01 |

**Table S9:** Test of heterochrony via variation in trajectory scaling (Gerber & Hopkins, 2011). A significant value indicates a significant difference in both trajectories in size-shape space and a rejection of the null hypothesis of heterochrony.

|  | *F. catenat* | *F. chrysot* | *F. confluen* | *F. diaph* | *F. escambi* | *L. goodei* | *F. grandis* | *P. guate* | *F. hetero* | *F. kansae* | *F. majalis* | *F. notat* | *F. nottii* | *F. olivace* | *L. parva* | *F. similis* | *F. stellifer* | *A. xenica* | *F. zebrinus* |
| --- | --- | --- | --- | --- | --- | --- | --- | --- | --- | --- | --- | --- | --- | --- | --- | --- | --- | --- | --- |
| *F. catenatus* | 1 |  |  |  |  |  |  |  |  |  |  |  |  |  |  |  |  |  |  |
| *F. chrysotus* | 0.0001 | 1 |  |  |  |  |  |  |  |  |  |  |  |  |  |  |  |  |  |
| *F. confluentus* | 0.0001 | 0.0001 | 1 |  |  |  |  |  |  |  |  |  |  |  |  |  |  |  |  |
| *F. diaphanus* | 0.0001 | 0.0001 | 0.0001 | 1 |  |  |  |  |  |  |  |  |  |  |  |  |  |  |  |
| *F. escambiae* | 0.0001 | 0.0001 | 0.0001 | 0.0001 | 1 |  |  |  |  |  |  |  |  |  |  |  |  |  |  |
| *L. goodei* | 0.0001 | 0.0001 | 0.0001 | 0.0001 | 0.0001 | 1 |  |  |  |  |  |  |  |  |  |  |  |  |  |
| *F. grandis* | 0.0001 | 0.0001 | 0.0001 | 0.0001 | 0.0001 | 0.0001 | 1 |  |  |  |  |  |  |  |  |  |  |  |  |
| *P. guatemalensis* | 0.0001 | 0.0001 | 0.0001 | 0.0001 | 0.0001 | 0.0001 | 0.0001 | 1 |  |  |  |  |  |  |  |  |  |  |  |
| *F. heteroclitus* | 0.0001 | 0.0001 | 0.0001 | 0.0001 | 0.0001 | 0.0001 | 0.0001 | 0.0001 | 1 |  |  |  |  |  |  |  |  |  |  |
| *F. kansae* | 0.0001 | 0.0001 | 0.0001 | 0.0001 | 0.0001 | 0.0001 | 0.0001 | 0.0001 | 0.0001 | 1 |  |  |  |  |  |  |  |  |  |
| *F. majalis* | 0.0001 | 0.0001 | 0.0001 | 0.0001 | 0.0001 | 0.0001 | 0.0001 | 0.0001 | 0.0001 | 0.0001 | 1 |  |  |  |  |  |  |  |  |
| *F. notatus* | 0.0001 | 0.0001 | 0.0001 | 0.0001 | 0.0001 | 0.0001 | 0.0001 | 0.0001 | 0.0001 | 0.0001 | 0.0001 | 1 |  |  |  |  |  |  |  |
| *F. nottii* | 0.0001 | 0.0001 | 0.0001 | 0.0001 | 0.0001 | 0.0001 | 0.0001 | 0.0001 | 0.0001 | 0.0001 | 0.0001 | 0.0001 | 1 |  |  |  |  |  |  |
| *F. olivaceus* | 0.0001 | 0.0001 | 0.0001 | 0.0001 | 0.0001 | 0.0001 | 0.0001 | 0.0001 | 0.0001 | 0.0001 | 0.0001 | 0.0001 | 0.0001 | 1 |  |  |  |  |  |
| *L. parva* | 0.0001 | 0.0001 | 0.0001 | 0.0001 | 0.0001 | 0.0001 | 0.0001 | 0.0001 | 0.0001 | 0.0001 | 0.0001 | 0.0001 | 0.0001 | 0.0001 | 1 |  |  |  |  |
| *F. similis* | 0.0001 | 0.0001 | 0.0001 | 0.0001 | 0.0001 | 0.0001 | 0.0001 | 0.0001 | 0.0001 | 0.0001 | 0.0001 | 0.0001 | 0.0001 | 0.0001 | 0.0001 | 1 |  |  |  |
| *F. stellifer* | 0.0001 | 0.0001 | 0.0001 | 0.0001 | 0.0001 | 0.0001 | 0.0001 | 0.0001 | 0.0001 | 0.0001 | 0.0001 | 0.0001 | 0.0001 | 0.0001 | 0.0001 | 0.0001 | 1 |  |  |
| *A. xenica* | 0.0001 | 0.0001 | 0.0001 | 0.0001 | 0.0001 | 0.0001 | 0.0001 | 0.0001 | 0.0001 | 0.0001 | 0.0001 | 0.0001 | 0.0001 | 0.0001 | 0.0001 | 0.0001 | 0.0001 | 1 |  |
| *F. zebrinus* | 0.0001 | 0.0001 | 0.0001 | 0.0001 | 0.0001 | 0.0001 | 0.0001 | 0.0001 | 0.0001 | 0.0001 | 0.0001 | 0.0001 | 0.0001 | 0.0001 | 0.0001 | 0.0001 | 0.0001 | 0.0001 | 1 |

**Table S10:** Test of heterochrony via disruption in the relationship between size and shape (Gerber & Hopkins, 2011). A significant value indicates a significant difference in both trajectories in shape space only and a rejection of heterochrony. The observed summed squared residuals were only found within the distribution of randomly generated summed squared residuals (i.e. leading to a non-significant difference between the observed value and the randomly generated distribution) on one occasion, between the species’ pair, *Fundulus kansae* and *Fundulus zebrinus*).

|  | *F. catenat* | *F. chrysot* | *F. confluen* | *F. diaph* | *F. escambi* | *L. goodei* | *F. grandis* | *P. guate* | *F. hetero* | *F. kansae* | *F. majalis* | *F. notat* | *F. nottii* | *F. olivace* | *L. parva* | *F. similis* | *F. stellifer* | *A. xenica* | *F. zebrinus* |
| --- | --- | --- | --- | --- | --- | --- | --- | --- | --- | --- | --- | --- | --- | --- | --- | --- | --- | --- | --- |
| *F. catenatus* | 1 |  |  |  |  |  |  |  |  |  |  |  |  |  |  |  |  |  |  |
| *F. chrysotus* | 0.002 | 1 |  |  |  |  |  |  |  |  |  |  |  |  |  |  |  |  |  |
| *F. confluentus* | 0.002 | 0.002 | 1 |  |  |  |  |  |  |  |  |  |  |  |  |  |  |  |  |
| *F. diaphanus* | 0.002 | 0.002 | 0.002 | 1 |  |  |  |  |  |  |  |  |  |  |  |  |  |  |  |
| *F. escambiae* | 0.002 | 0.002 | 0.002 | 0.002 | 1 |  |  |  |  |  |  |  |  |  |  |  |  |  |  |
| *L. goodei* | 0.002 | 0.002 | 0.002 | 0.002 | 0.002 | 1 |  |  |  |  |  |  |  |  |  |  |  |  |  |
| *F. grandis* | 0.002 | 0.002 | 0.002 | 0.002 | 0.002 | 0.002 | 1 |  |  |  |  |  |  |  |  |  |  |  |  |
| *P. guatemalensis* | 0.002 | 0.002 | 0.002 | 0.002 | 0.002 | 0.002 | 0.002 | 1 |  |  |  |  |  |  |  |  |  |  |  |
| *F. heteroclitus* | 0.002 | 0.002 | 0.002 | 0.002 | 0.002 | 0.002 | 0.002 | 0.002 | 1 |  |  |  |  |  |  |  |  |  |  |
| *F. kansae* | 0.002 | 0.002 | 0.002 | 0.002 | 0.002 | 0.002 | 0.002 | 0.002 | 0.002 | 1 |  |  |  |  |  |  |  |  |  |
| *F. majalis* | 0.002 | 0.002 | 0.002 | 0.002 | 0.002 | 0.002 | 0.002 | 0.002 | 0.002 | 0.002 | 1 |  |  |  |  |  |  |  |  |
| *F. notatus* | 0.002 | 0.002 | 0.002 | 0.002 | 0.002 | 0.002 | 0.002 | 0.002 | 0.002 | 0.002 | 0.002 | 1 |  |  |  |  |  |  |  |
| *F. nottii* | 0.002 | 0.002 | 0.002 | 0.002 | 0.002 | 0.002 | 0.002 | 0.002 | 0.002 | 0.002 | 0.002 | 0.002 | 1 |  |  |  |  |  |  |
| *F. olivaceus* | 0.002 | 0.002 | 0.002 | 0.002 | 0.002 | 0.002 | 0.002 | 0.002 | 0.002 | 0.002 | 0.002 | 0.002 | 0.002 | 1 |  |  |  |  |  |
| *L. parva* | 0.002 | 0.002 | 0.002 | 0.002 | 0.002 | 0.002 | 0.002 | 0.002 | 0.002 | 0.002 | 0.002 | 0.002 | 0.002 | 0.002 | 1 |  |  |  |  |
| *F. similis* | 0.002 | 0.002 | 0.002 | 0.002 | 0.002 | 0.002 | 0.002 | 0.002 | 0.002 | 0.002 | 0.002 | 0.002 | 0.002 | 0.002 | 0.002 | 1 |  |  |  |
| *F. stellifer* | 0.002 | 0.002 | 0.002 | 0.002 | 0.002 | 0.002 | 0.002 | 0.002 | 0.002 | 0.002 | 0.002 | 0.002 | 0.002 | 0.002 | 0.002 | 0.002 | 1 |  |  |
| *A. xenica* | 0.002 | 0.002 | 0.002 | 0.002 | 0.002 | 0.002 | 0.002 | 0.002 | 0.002 | 0.002 | 0.002 | 0.002 | 0.002 | 0.002 | 0.002 | 0.002 | 0.002 | 1 |  |
| *F. zebrinus* | 0.002 | 0.002 | 0.002 | 0.002 | 0.002 | 0.002 | 0.002 | 0.002 | 0.002 | ***0.052*** | 0.002 | 0.002 | 0.002 | 0.002 | 0.002 | 0.002 | 0.002 | 0.002 | 1 |
