# Supplementary material for: Does Body Shape in Fundulus Adapt to Variation in Habitat Salinity?
Source: Front Physiol. 2019 Nov 15;10:1400. doi: 10.3389/fphys.2019.01400 (PMC6872640; doi:10.3389/fphys.2019.01400)
Supplement: Supplementary file 8 [file Table_2.DOCX]

**Description A1: Description of landmark locations on *Fundulus***

The configuration of landmarks (Figure 1 in main text) mirrored that of Schaefer and Duvernell (2011), with two minor exceptions: i) elimination of second landmark (LM) and ii) movement of the 5^th^ landmark to the center of the eye. LM1 was located at the most anterior point on the snout; LM2 at the insertion of the check scale; LM3 at the dorsal insertion of the opercle at the most dorsal region of the body; LM4 at the origin of the dorsal fin; LM5 at the insertion of the dorsal fin; LM6 at the dorsal most, posterior insertion of the caudal fin; LM7 at the dorsal end of the caudal peduncle; LM8 at the ventral most, posterior insertion of the caudal fin; LM9 at the insertion of the anal fin; LM10 at the origin of the anal fin; LM11 at the ventral insertion of the opercle; LM12 at the ventral insertion of the cheek scale; LM13 was placed in the middle of LM3 and LM4 and was considered a semi-landmark; LM14 was placed in between LM10 and LM11 and was considered a semi-landmark. Both of these LMs were permitted to ‘slide’ to minimize the bending energy of curves (Zeldtich et al., 2004). LM16 was placed in the center of the eye.

**Description A2: Calculating the surface area of the opercular epithelium and gill arch length**

To determine whether the surface area of the opercular epithelium was significantly predicted by salinity niche, we focused on first calculating linear distances around LMs 2, 3, 16, 13, and 14. We divided this area into two geometric shapes: a triangle (LMs 2, 3, and 16) and a trapezoid (LMs 2, 16, 14 and 13) to ease our estimation of surface area. To estimate the area of the triangle, we first estimated the midpoint (below called ‘LM 17’) between LMs 2 and 16 as (A4.1):

$LM17=(\frac{X_{2}+X_{16}}{2}, \frac{Y_{2}+Y_{16}}{2})$

We then measured the height of the triangle as the distance between LM 17 and LM 2 using the following equation (A4.2):

$${\surd((X17-X2)}^{2}+{(Y17-Y2)}^{2})$$

The area of the triangle was then taken as ½*base*height with the base being the distance between LM 2 and 16. To measure the height of the trapezoid, we first calculated the midpoint between LM 2 and 14 using equation A4.1. Next, we found the area of the trapezoid using the following equation:

($\frac{Distance between LM 2 and 16+Distance between LM 2 and 14}{2})*height of trapezoid$

The surface area of the opercular epithelium was then determined by summing the area of the triangle with the area of the trapezoid. Gill arch length (mm) was calculated as the distance between LM13 and LM16.

**Description A3:** Description of Univariate (SLOUCH) and Multivariate Stochastic Linear Ornstein-Uhlenbeck models for Comparative Hypotheses (mvSLOUCH)

SLOUCH (Stochastic Linear Ornstein-Uhlenbeck models for Comparative Hypotheses, Hansen et al., 2008) and mvSLOUCH (Bartoszek et al., 2012) are flexible modeling packages implemented in R statistical and computing environment that allow one to model the evolution of a trait value (or suite of trait values) across a set of phylogenetically related species in response to various hypothesized selective regimes. In the univariate case, SLOUCH allows one to model shifting hypothesized, environmentally-determined trait optima on a phylogeny as i) fixed factors with any number of levels where past changes in optima can be mapped onto the phylogeny using any of the ancestral state reconstruction techniques (e.g. parsimony) – see also Butler and King (2004); ii) any number of fixed covariates (useful for modeling phenomenon such as mechanical allometric relationships); iii) any number of randomly evolving covariates where the underlying model of change is assumed to be Brownian motion determined by its own mean and variance parameters; and iv) any combination of the above. The only limitation is that analytical procedures for dealing with interaction terms have not yet been developed.

These models can be implemented in a likelihood framework, where various information criteria (e.g. AICc) are used to quantify the support (penalized by parameter numbers) for various alternate hypotheses related to the evolution of a trait. Trait evolution by Brownian motion, which represents a null model where evolution is neutral with respect to the hypothesized selective regimes, can also be included in this framework. We include supplementary tables that describe: the parameters estimated by the univariate SLOUCH models implemented in this paper along with their interpretations (Supplementary Table S2); and the model selection results for each landmark modeled as Brownian motion and one of the three salinity niche measurements: salinity scope (differences between highest and lowest salinity tolerance), maximum salinity, and average salinity tolerance (Supplementary Table S3). For more information about salinity tolerance measures and species salinity tolerances, see Ghedotti and Davis (2013). We then provide a similar table for the multivariate extensions, along with the results of landmark combinations modeled in this framework (Supplementary Table S4) and finally provide the full estimates for both univariate and multivariate models that were used to inform each other (Supplementary Table S8). Like all phylogenetic comparative methods, SLOUCH seeks to correct for statistical non-independence (pseudoreplication) that potentially arises from comparing data across species that share common ancestors. Most existing comparative methods assume that phylogenetic relatedness always introduces non-independence, the degree of which is determined solely by the ratio of branch lengths till the last common ancestor, and the common ancestral branch length to the root of the tree (so called phylogenetic effects, reviewed in Hansen and Orzack, 2005). The SLOUCH framework does not assume non-independence whenever there is phylogenetic structure but rather attempts to estimate a rate of adaption parameter (Supplementary Table S2) that can be used to modulate the degree of non-independence between trait values. It is the use of an Ornstein-Uhlenbeck process, with both deterministic and purely stochastic terms as the underlying model of trait evolution, that allows for this. If the rate of adaption of trait values is fast, phylogenetic structure does not matter. If, however, it is estimated to be a long slow process, shared common ancestry will be a major predictor of current trait values, and this source of non-independence (phylogenetic inertia) should then be corrected for. The parameters that encapsulate this modeling approach are described in Supplementary Table S2. The multivariate extension of SLOUCH, mvSLOUCH allows the user to examine various coadaptation scenarios between traits as they adapt to each other as well as to fixed or randomly evolving components of the environments. The model framework is well suited to modeling three coadaptation scenarios: i) correlated adaptation for traits that need to function in a synchronized manner; ii) trade-offs between traits under limited resource conditions; iii) and various allometric relationships between traits. Our hypotheses for body shape evolution correspond most closely to the first scenario, where aspects of body shape synchronize their evolution in response to the hypothesized trait optima set by salinity niches. The maximum number of response traits we could model simultaneously with the number of species we have, and still recover reasonable parameter estimates, was two. We therefore adopted the approach of testing pairs of landmark values that were either on opposite sides (dorsal vs. ventral) of the fish in the Y plane, or adjacent to each other on the X plane. The idea is that if the distance between landmarks increases systematically and at the same rate in the Y plane, the overall result is a robust body, and we can infer selection for increased body robustness. This may come at the cost of having to reduce the distance between points in the X plane (overall size remains the same). The converse may also be true, in which case selection is favoring longer, skinnier fish.

For the multivariate traits modeled, we estimate a matrix of parameter values for some of the parameters listed in Supplementary Table S2. For example, whereas a single α estimate describes the rate of evolution for a given trait, for two traits modeled simultaneously we estimate the following matrix of α values:

$$\begin{matrix} \alpha_{11} & \alpha_{12} \\ \alpha_{21} & \alpha_{22} \end{matrix}$$

Similarly, we require a matrix of values for the stochastic variance and covariance associated with each trait. It is easy to see that we quickly run into very large numbers of parameters that need to be estimated for the multivariate models (e.g. we add 6 additional parameters for 2 traits, whereas for three traits it would be 18 additional parameters on top of those that already need to be estimated). Given the limited size of our data set, we need to make some simplifying assumptions to estimate parameters with any degree of confidence. One way we can do this is to assume a single α and single σ^2^_Y_ value that jointly describes the bivariate evolution of a set of two landmarks. We can provide some justification for this approach by comparing the univariate estimates for α (or rather its converse) modeled on the same environmental variables to each other for a given pair of traits – if they are roughly the same (relative to the overall parameter space), then this provides evidence (admittedly qualitative) that they are adapting to the environment at the same rate. First, we provide the model selection results for the multivariate landmark pairs modeled on salinity scope as a predictor variable, as compared to the multivariate Brownian motion model of evolution (neutrality with respect to scope).

**How do the univariate and multiple model selection results compare?**

Salinity Scope is the predictor that most often results in the best model for the evolution of a given landmark (22 of the 32 landmark variables), followed by a Brownian motion model (6 of the 32 variables), then Max Salinity (3 of the 32 landmarks), and lastly average salinity (1 of the 32 variables)(Supplementary Table S3). We therefore focused the multivariate analyses on Salinity Scope. The univariate landmarks best described by Scope are X2, X3, X6, X10, X11, X12, X14, X15, X16 and Y1, Y2, Y3, Y4, Y5, Y6, Y9, Y10, Y11, Y12, Y13, Y14 and Y15 (Supplementary Table S3). For the multivariate models, the combinations of landmarks that performed best under a model including Scope were Y3-Y11 (both also picked up in the univariate models), Y6-Y8 (Scope the best for Y6 but Average Salinity the best for Y8 in univariate models), X2-X3 (both also picked up in the univariate models), X4-X5 (both predicted to be best described by a Brownian motion model in the univariate case). For X10-X11 and X11-X12, all three landmarks were also picked up in the univariate model as being affected by Salinity Scope. Thus, besides X4 and X5, which were picked up by the multivariate model as being best described by Salinity Scope rather than a Brownian motion, all other landmarks picked up by the multivariate model were also picked up in the univariate models as best described by Salinity Scope. That several landmarks that were best described by salinity scope in the univariate models were best described by a Brownian motion in the multivariate model probably reflects the much heavier parameter number penalization that the multivariate adaptive models undergo as compared to the univariate or multivariate Brownian motion models. Lastly, whereas most multivariate landmark pairs had a joint half-life estimate within the ballpark range of their individual univariate half-life estimates as well as being relatively small compared to overall phylogenetic tree height (30% or less), this was not the case for X11 and X12 which had a joint estimate of 1.13 (larger than the overall tree height). We therefore excluded this landmark pair from further interpretation as the assumption is that shape (as captured by landmarks), responds to selection so only those landmark pairs that respond with similar rates of adaptation are interpretable in the context of our hypothesis. We note that confidence intervals are extremely wide for all multivariate estimates, but also that the lower limit confidence interval for X11-X12 does not include the half-life estimate for any of the other landmark pairs. Taken together with the univariate analysis, we are confident in concluding that certain landmark pairs respond to selection pressures imposed by salinity scope, and they do so as part of a multivariate shape response.

**What can we infer from the parameter estimates?**

So far we have not discussed the parameters that predict optimal (expected) and evolutionary regressions describing the relationship between traits and the environmental variables. We focus here on the multivariate pairs of landmarks that were best described by Salinity Scope: Y3-Y11, Y6-Y8, X2-X3, X4-X5, X9-X10, X10-X11, excluding X11-X12 as discussed above. The optimal partial regression parameters for intercepts and slopes for each landmark in the above-mentioned multivariate analyses are given in Supplementary Table S5. These were used to generate Figure 5 in the manuscript as described. The smallest value for Scope was 0.025 and the largest value 84.5 – these were multiplied by the regression coefficients to obtained predictions for the expectations of these landmark positions under instantaneous adaptation.

**Description A4: Test of heterochrony**

Although developmental change can take on a variety of recognized forms (Zelditch et al., 2000), the change in the rate or timing of development has been argued as one of the most important sources of variation upon which selection acts to drive evolutionary change (Gould, 1977; Alberch et al., 1979). Many studies that compare developmental sequences, and patterns of gene expression underlying the development of specific anatomical characteristics among species have shown that changes in the rate or timing of development, most often mechanistically attributed to heterochrony, can have important implications for morphological evolution (Mabee et al., 2000; Goswami, 2007; Albertson et al., 2010; Tills et al., 2011).

When comparing ontogenetic trajectories constructed with multivariate shape data, it is possible to study heterochrony between species. However, this is not always straightforward because any change in the location of one species’ trajectory relative to another can be viewed as heterochrony, even when both trajectories never shared a similar location in multivariate shape space (i.e. morphospace; Mitteroecker et al., 2005). Because ‘classical’ heterochrony as described by Gould (1977) and Alberch et al. (1979) requires that ontogenetic trajectories overlap at some point in development and diverge elsewhere, describing all differences in ontogenetic trajectories among species as heterochrony is, in the classical sense, incorrect. As a result, many techniques have been devised to explicitly test for ‘classic’ multivariate heterochrony (Gerber and Hopkins, 2011; Cvijanovic et al., 2014; Piras et al., 2011; Piras et al., 2014; Piras et al., 2016). In addition, considering the theoretical and empirical importance of heterochrony in morphological evolution (Keyte and Smith, 2014), we hypothesize that ‘classic’ heterchrony should explain significant morphological diversification and evolution within the genus.

**Test for ‘Classic’ Heterochrony**

Two permutation tests provided by Gerber and Hopkins (2011) were used to test for ‘classic’ multivariate heterochrony between each species pair. The first was used to test for multivariate heterochrony via variation in trajectory scaling, such that two species’ trajectories occupy a similar size-shape space, but are either truncated or extended relative to one another. The second was used to test for multivariate heterochrony via disruption between size and shape, such that both species exhibit similar shape variation in their respective developmental trajectory (i.e. trajectories occupy the same shape space), but not necessarily at a similar time (or size) in development (i.e. trajectories occupy different size-shape space). The first test of heterochrony calculates the within-species summed squared residuals of a multivariate regression of shape on size using the correct species designation for each specimen (Gerber et al., 2007). The test then randomly assorts specimens to species, and then recalculates the within-species summed squared residuals over many hundred iterations. From this, the test creates a distribution in which to compare the observed within-species summed squared residuals. For the hypothesis of heterochrony to be accepted, the observed within-species summed squared residuals must lie within this distribution, implying that the species’ trajectories are close together. The second test is essentially the same as the first, except the sum of squared distances from each specimen to the predicted shape on the multivariate regression is used as the test statistic (Mitteroecker et al., 2005). Since exact age data were not available for the specimens included in this study, the specific type of multivariate heterochrony among species (i.e. paedomorphosis or peramorphosis) could not be identified because dissociation of size, shape, and age is required to distinguish between paedomorphosis and peramorphosis (Gerber and Hopkins, 2011).

**Heterochrony in *Fundulus***

Heterochrony was not found to be driving the evolution of morphological variation in *Fundulus*. Of the 342 pairwise permutation tests run among the 19 species of *Fundulus*, the observed summed squared residuals were only found within the distribution of randomly generated summed squared residuals on one occasion, between the species pair *Fundulus kansae* and *Fundulus zebrinus* (see Tables S6 and S7). This means that the hypothesis of heterochrony (specifically, via variation in trajectory scaling) could only be accepted 0.29% of the time in *Fundulus* species observed here. All other pairwise comparisons revealed that the ontogenetic trajectories for each species pair were distinct.

We aimed to first determine if ‘classic’ heterochrony (Gould, 1977) could in part explain the evolution of ontogenetic trajectories among *Fundulus*. Of the 342 pairwise tests of heterchrony, we found only a single instance of multivariate heterochrony (i.e. via ontogenetic scaling) in *Fundulus*. Thus, the vast majority of variance in the ontogenetic trajectories between species within the genus can be attributed to ‘allometric repatterning’ (Gerber et al., 2007), or the evolution of distinguishable ontogenetic trajectories between species. Considering the strong theoretical basis of ‘classic’ heterochrony as an important contributor to morphological evolution (Gould, 1977; McNamara, 1997; Richardson et al. 2009), and many examples of heterochrony among other species (Gerber et al., 2007; Gerber and Hopkins, 2011), it is surprising that we found only one instance within the genus *Fundulus*. It is possible that we may have underestimated the ability of selection to generate considerable variation in ontogenetic body shape trajectories over macroevolutionary time, since there can be many adaptive pathways that lead to a single fitness optimum (Ord et al., 2015). For instance, although several species of cave salamanders develop similar, high surface area foot morphologies, well suited for climbing at the adult stage, the developmental trajectory that leads them to that phenotype can be very different (Adams and Nistri, 2010).

References

Adams, D. C., and Nistri, A. (2010). Ontogenetic convergence and evolution of foot morphology in European cave salamanders (Family: Plethodontidae). BMC Evol. Biol. 10, 216.

Alberch, P. 1979. Size and shape in ontogeny and phylogeny. Paleobiology. 5, 296-317.

Albertson, R.C., Y-L. Yan, T.A Titus, E. Pisano, M. Vacchi, P.C. Yelick, H.W. Detrich III, and Postlethwait, J.H. (2010). Molecular pedomorphism underlies craniofacial skeletal evolution in Antarctic notothenioid fishes. BMC Evol. Biol. 10, 4.

Bartoszek, K., Pienaar, J., Modstad, P., Andersson, S., and Hansen, T. F. (2012). A phylogenetic comparative method for studying multivariate adaptation. J. Theor. Biol. 314, 204–215.

Butler, M. A., and King, A. A. (2004). Phylogenetic comparative analysis: a modeling approach for adaptive evolution. Am. Nat. 164, 683–695.

Cvijanović, M., Ivanovic, A. Kolarov, A., Džukic, G., and Kalezić, M.L. (2009). Early ontogeny shows the same interspecific variation as natural history parameters in the crested newt (Triturus cristatus superspecies) (Caudata, Salamandridae). Contributions of Zoology, 78, 43- 50.

Gerber, S., Neige, P., and Eble, G.J. (2007). Combining ontogeny and evolutionary scales of morphological disparity: a study of early Jurassic ammonites. Evol. Dev. 9, 475-482.

Gerber, S., and Hopkins, M. J. (2011). Mosaic heterochrony and evolutionary modularity: The trilobite Genus *Zacahthopsis* as a case study. Evolution. 65, 3241-3252.

Ghedotti, M. J., and Davis, M. P. (2013). Phylogeny, classification, and evolution of salinity tolerance of the North American topminnows and killifishes, Family Fundulidae (Teleostei: Cyprinodontiformes). Fieldiana Life Earth Sci. 7, 1–65.

Goswami, A. (2007). Cranial modularity and sequence heterochrony in mammals. Evol. Dev. 9, 290-298.

Gould, S. J. 1977. Ontogeny and Phylogeny. Harvard Univ. Press, Cambridge, MA.

Mabee, P.M., Olmstead, K.L., and Cubbage, C.C. (2000). An experimental study of intraspecific variation, developmental timing, and heterochrony in fishes. Evolution. 54, 2091-2106.

McNamara, K. J. 1997. Shapes of Time: The evolution of growth and development. John Hopkins Univ. Press, Baltimore, Maryland.

Mitteroecker, P., P. Gunz and F.L. Bookstein. 2005. Heterochrony and geometric morphometrics: a comparison of cranial growth in *Pan paniscus* versus *Pan troglodytes*. Evol. Dev. 7:244–258.

Ord, T.J., Klomp, D.A., Garcia-Porta, J., and Hagman, M. (2015). Repeated evolution of exaggerated dewlaps and other throat morphology in lizards. J. Evol. Biol. 28, 1948-1964.

Piras, P., Salvi, D., Ferrara, G., Maiorino, L., Delfino, M., Pedde, L., and Kotsakis, T. (2011). The role of post-natal onotgeny in the evolution of phenotypic diversity in Podarcis lizards. J. Evol. Biol. 24, 2705-2720.

Piras, P., Evangelista, A., Gabriele, S., Nardinocchi, P, Teresi, L., Torromeo, C., Schiariti, M., Varano, V., and Puddu, P.E. (2014). 4-D analysis of left ventricular heart cycle using Procrustes motion analysis. PLoS ONE 9, e86896.

Piras, P., Teresi, L., Traversetti, L., Varano, V., Gabriele, S., Kotsakis, T., Raia, P., Puddu, P.E., and Scalici, M. (2016). Conceptual framework of ontogenetic trajectories: parallel transport allows recognition and visualization of pure deformation patterns. Evol. Devel. 18, 182-200.

Richardon, M.K., Gobes, S.M.H., van Leeuwen, A.C., Polman, J.A.E., Pieau, C., and Sánchez-Villagra, M.R. (2009). Heterochrony in limb evolution: Developmental mechanisms and natural selection. J. Exp. Zool. 312B, 639-664.

Schaefer, J., and Duvernell, D. (2011). Shape variability in topminnows (*Fundulus notatus* species complex) along the river continuum. Biol. J. Linn. Soc 103, 612-621.

Tills, O., Rundle, S.D., and Spicer, J.I., (2013). Variance in developmental event timing is greatest at low biological levels: implications for heterochrony. Biol. J. Linnean. Soc. 110, 581.590.

Zeldtich, M.L., Sheets, H.D., and Fink, W. L. (2000). Spatiotemporal reorganization of growth rates in the evolution of ontogeny. Evolution. 54, 1363-1371.
